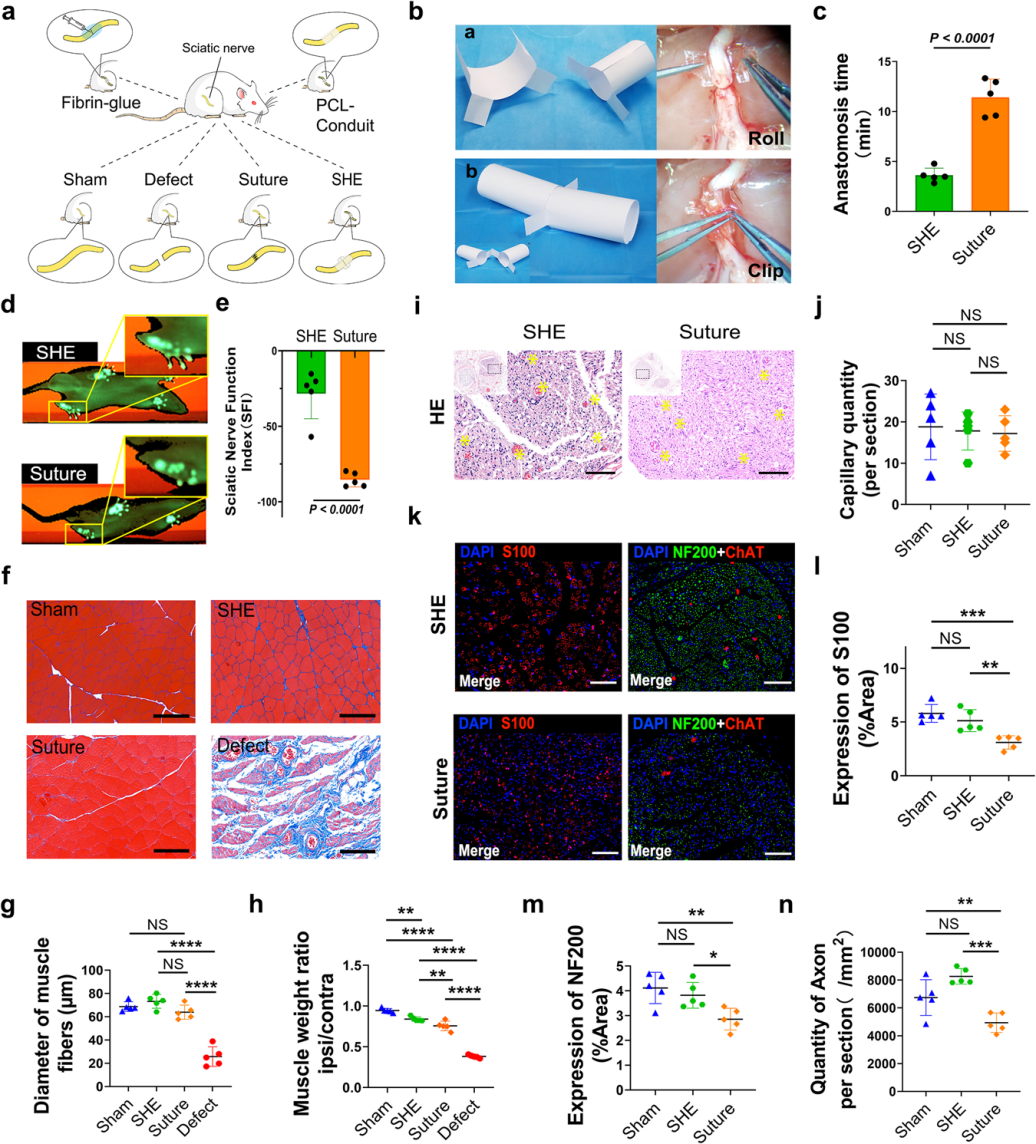# Publisher Correction: Self-healing polyurethane-elastomer with mechanical tunability for multiple biomedical applications in vivo

**DOI:** 10.1038/s41467-022-28166-2

**Published:** 2022-05-05

**Authors:** Chenyu Jiang, Luzhi Zhang, Qi Yang, Shixing Huang, Hongpeng Shi, Qiang Long, Bei Qian, Zenghe Liu, Qingbao Guan, Mingjian Liu, Renhao Yang, Qiang Zhao, Zhengwei You, Xiaofeng Ye

**Affiliations:** 1 Department of Cardiovascular Surgery, Ruijin Hospital, Shanghai Jiao Tong University, School of Medicine, Shanghai, China; 2 State Key Laboratory for Modification of Chemical Fibers and Polymer Materials, Shanghai Belt and Road Joint Laboratory of Advanced Fiber and Low-dimension Materials (Donghua University), College of Materials Science and Engineering, Donghua University, Shanghai, China; 3 Department Neuro Surgery, Ruijin Hospital, Shanghai Jiao Tong University, School of Medicine, Shanghai, China; 4 Department Orthopedics Surgery, Ruijin Hospital, Shanghai Jiao Tong University, School of Medicine, Shanghai, China

**Keywords:** Vascular diseases, Surgery, Peripheral neuropathies, Biomedical materials

Correction to: *Nature Communications* 10.1038/s41467-021-24680-x, published online 20 July 2021.

Fig. 4 was missing from the PDF version of this article; the figure should have appeared as shown below. The original article PDF has been corrected. The HTML version was unaffected.