# Supplementary material for: Self-healing polyurethane-elastomer with mechanical tunability for multiple biomedical applications in vivo
Source: Nat Commun. 2021 Jul 20;12:4395. doi: 10.1038/s41467-021-24680-x (PMC8292539; doi:10.1038/s41467-021-24680-x)
Supplement: Supplementary file 1 — Supplementary Information [file 41467_2021_24680_MOESM1_ESM.pdf]

Supplementary Information for

The Self-healing Polyurethane-elastomer with Mechanical Tunability for Multiple Biomedical Applications in vivo

Chenyu Jiang, Luzhi Zhang, Qi Yang, Shixing Huang, Hongpeng Shi, Qiang Long, Bei Qian, Qiang Zhao*, Zhengwei You*, Xiaofeng Ye*

***Corresponding author**:
Email: zq11607@rjh.com.cn; zyou@dhu.edu.cn; xiaofengye@hotmail.com

Supplementary Methods

Evaluate SHEs biocompatibility in vitro

Considering the fact that direct contact of target tissue to the SHEs is the outer membrane composed of fibroblasts, the primary mouse areolar fibroblast was used in this part of study. Followed the reported culture protocol, areolar fibroblasts originated from mouse tail dermal tissue were expanded for two passages before testing. Fibroblasts were seeded on the 96-well plates (3×10^3 cells per well). Two kinds of elastomers were used including elastomer with catalyst which we previously synthesized as a negative control and SHE2. Besides, PCL, which was approved by FDA for medical apparatus fabrications[1], was used as a positive control. The materials were placed above cell in the well after adherence of fibroblasts. The Cell Counting Kit-8 (CCK-8) (Dojindo Laboratories, Kumamoto, Japan) was used as previously reported to verify the biocompatibility of material on the hours of 6, 12, 24, 48, 72 of post-seeding respectively. Briefly, 10 μL of CCK-8 was added to the wells contained with 100 μL of MEM culture medium (11095-080, Gibco, ThermoFisher) and incubated at 37 °C for 45 min prior to measuring the absorbance values at 450 nm via spectrophotometer[2]. Cell proliferation rate was calculated following the instructions of product manual. Before live/dead cell staining (04511, sigma-aldrich), the cell slides were put on the bottom of 24-well plates and seeded with areolar fibroblast prior to co-culture with SHE2. On the hours of 24, 48, 72 of post-seeding, the staining was performed respectively according to manual instructions of kit and the stained cell slides were imaged under microscope (bx51; Olympus, Shinjuku, Tokyo, Japan).

In vivo degradation of SHEs and evaluation of SHEs side effects

SHEs were dried and weighted before implanted subcutaneously on the back of C57BL/6J mice. Moreover, for the part of evaluation of SHEs side effects, c-SHEs was used as a negative control in Ly6G analysis (acute inflammation analysis) and PCL was used as a positive control in F4/80 analysis (chronic inflammation analysis). Animals were sacrificed at 5, 10, 15, 25, 35 days after implantation. To illustrate the *in vivo* degradation of SHEs, the surface of SHE sheets on day 35 post-implantation were imaged by SEM (SU8010, Hitachi, Tokyo, Japan). Meanwhile, the skins of back were harvested for inflammatory analysis. Harvested tissues were fixed in 4% paraformaldehyde for 24 h before embedding in paraffin for cross section preparation. Paraffin sections were needed for immunostaining using primary antibodies of Ly6G (1:100, ab25377, Abcam) and F4/80 (1:100, ab90247, Abcam) to image the inflammation response on the skin. All stained slides were imaged under the automatic microscope. All examinations were conducted by 2 trained, independent observers blinded to the genotype and treatment. A mean value was determined from at least 4 sections from each animal. Intracardiac puncture by syringe filled with EDTA was done to collect the blood sample to verify the side effects of SHEs on the implanted mice, including liver function (alanine aminotransferase (ALT), aspartate aminotransferase (AST), direct bilirubin (DBIL), γ-glutamyl transpeptidase (γ-GT)), renal function (blood urea nitrogen (BUN) and serum creatinine (SCr))[3,4].

Limiting the progression of abdominal aortic aneurysm and corresponding result evaluation

According to the previous literatures[5-8], the aneurysm in this study was established by PPE adventitial application. It has been reported in the literature that there is no difference between PPE adventitial application and PPE infusion in the aneurysm formation time during the process of mouse aneurysm modeling[9]. And the pathological changes of aneurysm model are similar in PPE infusion and PPE adventitial application including the phenomenon of elastin degradation[10], macrophage infiltration[5], and smooth muscle apoptosis[11], which are also the major mechanisms of human abdominal aortic aneurysm pathogenesis[12]. The abdominal aorta above the renal artery in mice has many important organ branches, hence, Angiotensin II infusion model cannot be used as an experimental model for external arterial wrapping in animals. Operation process: briefly, after anesthesia and careful median laparotomy, the abdominal aorta was exposed from the renal vein to the iliac bifurcation before wrapped circumferentially by bibulous paper soaked with 10 μl of 100% elastase for 10 min and 0.9% saline flushing to stop reaction of elastase. In each SHEs groups, the SHEs were putted under abdominal artery before bended and wrapped the vessel until the edge of SHEs was healed together. In silicone rubber group (non-self-healing material control group), the rubber was putted under abdominal aorta and wrapped around aorta followed by suturing to stitch

edge of rubber together. To strength the claim of SHEs' efficiency on aneurysm limiting, we also wrapped the SHE1 and SHE0.5 on established aneurysm models. 3-day post aneurysm establishment is the time point of rapid aneurysm progress. We performed the SHEs (including SHE0.5 and SHE1) wrapping on the third day after establishing of aneurysm model. The in vivo images of enlarging aneurysm were captured before wrapping operation. *In vivo* aortic MRI was executed by a Bruker BioSpec 70/20 USR 7.0 T (Bruker, Ettlingen, Germany) imaging system equipped with microimaging gradients. Animals received anesthesia induction by isoflurane at 3% before imagination. During the MRI operations, 1.8% isoflurane with 300 mL/min air-flow was administered at physiologic conditions. Breath-monitoring and gating were obtained using a SA Instruments (Stony Brook, New York, USA) system and a pressure sensor connected to an air-filled balloon. Temperature was maintained at 37°C by heating of the surrounding magnetic field gradient coils. The detail parameter settings of MRI were followed by principles reported previously[13]. By flowing void effect of MRI in the cross section, the aorta will be illustrated as high signal region which is the structure of vessel lumen. The scan area of the abdominal aorta will be determined through the scout scanning before formal horizontal scanning. Using the sagittal image from the scout scanning to observe the shape of the abdominal aorta and determine the final positions of subsequent horizontal scanning, which ensures the image of the maximum dilation part of the abdominal aorta can be captured. The MRI scanning of each animal starts below the opening of the renal artery and ends at the bifurcation of the iliac artery. The length of the total scan segment is 15mm. After the mice were euthanized, left cardiac ventricles were perfused with phosphate-buffered saline followed by 4% paraformaldehyde via a syringe and catheter inserted into the left ventricle. A syringe (5ml) was used to inject liquid at a rate of 0.1ml per second. After the measurement of the electronic pressure gauge, the pressure produced by this bolus rate is about 100mmHg, which is similar to the arterial blood pressure of normal mice. These precautions can prevent blood vessels from collapsing or over-expanding. The arteries were then dissected. Harvested tissues were fixed in 4% paraformaldehyde for 24 h before embedding in paraffin for cross section preparation. HE staining: The paraffin-free sections washed by distilled water had been placed in an aqueous solution of hematoxylin for several minutes. Then, the sections were placed in acid and ammonia water respectively for separate color few seconds before washed by running water and soaked with distilled water. After dehydrated in 70% and 90% alcohol for 10 min each, the sections were placed into the alcohol eosin staining solution for 2-3 min. EVG staining: Serial sections were stained by using the elastica van gieson (EVG) staining kit following the manufacturer's protocol (No. 115974; EMD Millipore, Burlington, MA) for elastin assessment. The elastin grading system is comprised of four criterions: grade1: <25% of the medial circumference

digested; grade2, 25%-50% of the medial circumference digested; grade3: 50%-75% of the medial circumference digested; grade4, 75%-100% of the medial circumference digested. In order to estimate the grade of elastin degradation objectively, the image of EVG staining is equally divided into four quadrants and we grade elastin degradation based on the number of quadrants occupied by the elastin fragmentation area. An average of 4 sections per mouse was counted from each aorta examined. IH staining: Paraffin sections were needed for immunostaining using the following primary antibodies of eNOS (1:100, ab76198, Abcam). eNOS was quantified and presented as percentage of positive area. All stainings were imaged under the automatic microscope (bx51; Olympus, Shinjuku, Tokyo, Japan). All examinations were conducted by 2 trained, independent observers blinded to the genotype and treatment. A mean value was determined from at least 4 sections from each animal. IF staining: For immunofluorescent analysis, paraffin sections were permeabilized with 0.3% TritonX-100 in PBS for 5 min and blocked with 5% fetal bovine serum and 10% donkey serum in PBS at room temperature for 1 h. The sections were stained with anti-collagen I (1:100, ab6308, Abcam), anti-collagen III (1:200, PA592066, Invitrogen), anti-caspase-3(43-7800, Invitrogen), anti-F4/80 (1:100, ab90247, Abcam), anti-CD3(1:10, ab135372, Abcam) and anti-CD31(1:100, MA3100, Invitrogen) at room temperature. Alexa Fluor 488 goat anti-mouse IgG (1:200, ab150117, Abcam) and Alexa Fluor 594 goat anti-rabbit IgG (1:200, A32740, Invitrogen) were used as the secondary antibodies, respectively. The slides incubated with PBS with free of primary antibodies were used as negative controls. Tissue sections were further stained with DAPI for 5 min before imaging. An average of 4 sections per mouse was counted from each aorta examined.

Total RNA was extracted from abdominal aorta samples using TRIzol (Invitrogen, USA) and was then reverse-transcribed to cDNA using qRT-PCR kit (Invitrogen, USA) according to the manufacturer's instruction. The qRT-PCR was carried out using SYBR1 Green Real time PCR Master Mix (Toyobo Co. Ltd., Osaka, Japan) under the condition of 95 °C for 10 min, and followed with 40 cycles of 95 °C for 15 s and 60 °C for 60 s. The mRNA level of GAPDH was used as an internal control, and the relative gene expression levels were calculated using the $2^{-D\Delta Ct}$ method. Each gene was analyzed in triplicate. The primer sequences were listed as follows: eNOS, S 5'-TGGACATCACTTCCCCGCCTA-3' and A 5'- TGCCACTGAAGGAAATTGCTC-3'; caspase-3, 5'-TGGAAAGCCGAAACTCTTCATCA-3' and A 5'-CCACGACCCGTCCTTTGAAT-3'; GAPDH, S 5'-CCTCGTCCCGTAGACAAAATG-3'and A5'-TGAGGTCAATGAAGGGGTCGT-3'

Sciatic nerve coaptation and corresponding result assessment.

In the peripheral nerve part, 260 ± 10 g SD male rats were used in this part. Animals were divided into six groups: sham group, defect group, suture group, fibrin-glue group, PCL conduit group and

SHE0.2 group. The details of nerve operations in sham, defect, suture, fibrin-glue and PCL conduit group were described in supplement file. In the sham group, sciatic nerve was exposed from the notch to the bifurcation on the left limb of SD rat and no more operation was performed. In the defect group, after exposing, the nerve was cut off quickly by micro-scissor to create nerve defect model. In the suture group, after amputation, epineural coaptation was carefully performed by 10-0 microsurgical suture (Alcon Laboratories, USA) under an operating microscope. In fibrin-glue group, after amputation of sciatic nerve, we relieved the tension between stumps by same method as in SHE group, then we aligned the stumps of nerve by avoiding distortion and injected 0.5ml fibrin glue (FIBRINGLURAAS® Fibrin Sealant, NUANCE BIOTECH INC, China) around anastomotic area based on previous reports[14,15]. In the PCL conduit group, after amputation of sciatic nerve, we relieved the tension between stumps by same method as in SHE group, then we inserted the two nerve stumps into conduit gently and without distortion before fixing the conduit with nerve by suture. The target nerve was harvested in each group on the week 6 post of coaptation for further histology analysis including hematoxylin-eosin (HE), Fluorogold (FG) staining, LuxolFastBlue (LFB) staining, Transmission electron microscope (TEM), Masson trichrome (MT) staining and immunofluorescence (IF) staining. The details of histology analysis were illustrated in supplement file. After the rats were euthanized, the nerves and gastrocnemius muscles were dissected. Harvested tissues were fixed in 4% paraformaldehyde for 24 h before embedding in paraffin for cross section preparation. Serial sections were analyzed using the following method. HE staining: The details of HE staining protocol were same as mentioned above. IF staining: After routine preparing of the nerve samples, the sections were stained with anti-NF200 (1:50, ab82259, Abcam), anti-S100β (1:100, ab52642, Abcam), anti–ChAt (1:100, ab181023, Abcam) and anti-caspase-3 (43-7800, Invitrogen) at room temperature. Alexa Fluor 488 goat anti-mouse IgG (1:200, ab150117, Abcam), Alexa Fluor 594 goat anti-rabbit IgG (1:200, ab150088, Abcam) and Alexa Fluor 594 goat anti-rabbit IgG (1:200, ab150088, Abcam) were used as the secondary antibodies, respectively. The slides incubated with PBS with free of primary antibodies were used as negative controls. Tissue sections were further stained with DAPI for 5 min before imaging. LFB staining: After routine preparing of the muscle samples, serial sections were stained by using the LFB staining kit according to the manufacturer's protocol. Retrograde labeling sampling method：according to the protocol, the powder of Fluoro-Gold was dissolved in 0.9% saline. For retrograde labeling of ganglia and spinal cord connected with sciatic nerve, the distal sciatic nerve was cut and dipped in aq. 5% solution of fluoro-gold. The survival time of animals was 4 days post of labeling. At the 4-day post of labeling, the animal was euthanized and then fixed with paraformaldehyde. We exposed the whole course of the sciatic nerve under the microscope and found the ganglion corresponding to the sciatic nerve. Meanwhile, with the help of rongeurs, we opened the lamina of the animal's spine to expose the spinal cord. The ganglions and spinal cord from L4-L6 were extracted by microscissor. The specimens of ganglions and spinal cord were frozen and sectioned after dehydration with sucrose in a dark room. The slices were visualized under the fluorescence microscope using a wide band ultraviolet (UV) excitation filter and positive neurons emitted yellow light when excited by UV light (wide band UV, excitation filter BP 340-380). MT staining: After routine preparing of the muscle samples, serial sections were stained by using the Masson trichrome staining kit according to the manufacturer's protocol (HT15, Sigma).

Total RNA was extracted from sciatic nerve samples using TRIzol (Invitrogen, USA) and was then reverse-transcribed to cDNA using qRT-PCR kit (Invitrogen, USA) according to the manufacturer's

instruction. The qRT-PCR was carried out using SYBR1 Green Real time PCR Master Mix (Toyobo Co. Ltd., Osaka, Japan) under the condition of 95 °C for 10 min, and followed with 40 cycles of 95 °C for 15 s and 60 °C for 60 s. The mRNA level of GAPDH was used as an internal control, and the relative gene expression levels were calculated using the $2^{-D\Delta Ct}$ method. Each gene was analyzed in triplicate. The primer sequences were listed as follows: caspase-3, S 5'-GGATTACCCTGAAATGGGCTTG-3' and A 5'- ACAGGTCCGTTCGTTCCAAAA-3'; GAPDH, S 5'- CTGGAGAAACCTGCCAAGTATG-3'and A 5'- GGTGGAAGAATGGGAGTTGCT-3'

Sternum immobilization and corresponding result assessment.

Briefly, the large animals (porcine) were anesthetized with an injection of propofol (1 mg/kg, I.V.) and then were intratracheally intubated and ventilated. Anesthesia was maintained with 1.0% isoflurane. Arterial pressure catheter and surface electrocardiogram were connected to the data acquisition (DAQ) system. After routine median sternotomy, the animals were scanned on the chest firstly by auto-spin C-arm x-ray machine (Siemens, Berlin, Germany) to image the fractured sternum on the transverse section. Then, the preshaped SHE2s (30mm × 30mm × 120mm) were traversed through intercostal muscles on both sides and two ends of SHE was fixed and healed by clipping of hemostatic forceps to close sternum. The second scanning of closed sternum was performed after surgery as mentioned above. X-ray: On the week 6 post of surgery, the anesthetized rats were fixed on the plastic table which was placed on the scanning window of C-arm-x-ray machine (General Electric Company, Boston, Massachusetts, USA). HE staining: The details of HE staining protocol were same as mentioned above. MT staining: The details of Masson staining protocol were same as mentioned above.

1. Sun, H., Mei, L., Song, C., Cui, X. & Wang, P. The in vivo degradation, absorption and excretion of PCL-based implant. *Biomaterials* **27**, 1735-1740 (2006).
2. Wang, S., *et al.* Tamoxifen inhibits fibroblast proliferation and prevents epidural fibrosis by regulating the AKT pathway in rats. *Biochemical and biophysical research communications* **497**, 937-942 (2018).
3. Zheng, D.W., *et al.* Phage-guided modulation of the gut microbiota of mouse models of colorectal cancer augments their responses to chemotherapy. *Nat Biomed Eng* **3**, 717-728 (2019).
4. Zheng, X.C., *et al.* Successively activatable ultrasensitive probe for imaging tumour acidity and hypoxia. *Nat. Biomed. Eng* **1**, 9 (2017).
5. Bhamidipati, C.M., *et al.* Development of a novel murine model of aortic aneurysms using peri-adventitial elastase. *Surgery* **152**, 238-246 (2012).

6. Li, J., *et al.* IL (Interleukin)-33 Suppresses Abdominal Aortic Aneurysm by Enhancing Regulatory T-Cell Expansion and Activity. *Arteriosclerosis, thrombosis, and vascular biology* **39**, 446-458 (2019).
7. Johnston, W.F., *et al.* Inhibition of Interleukin-1 Decreases Aneurysm Formation and Progression in a Novel Model of Thoracic Aortic Aneurysms. *Circulation* **130**, S51-S59 (2014).
8. Lareyre, F., *et al.* TGFβ (Transforming Growth Factor-β) Blockade Induces a Human-Like Disease in a Nondissecting Mouse Model of Abdominal Aortic Aneurysm. *Arteriosclerosis, thrombosis, and vascular biology* **37**, 2171-2181 (2017).
9. Busch, A., *et al.* Extra- and Intraluminal Elastase Induce Morphologically Distinct Abdominal Aortic Aneurysms in Mice and Thus Represent Specific Subtypes of Human Disease. *J Vasc Res* **53**, 49-57 (2016).
10. Golledge, J., Krishna, S.M. & Wang, Y. Mouse models for abdominal aortic aneurysm. *Br J Pharmacol* (2020).
11. Gao, F., *et al.* Disruption of TGF-β signaling in smooth muscle cell prevents elastase-induced abdominal aortic aneurysm. *Biochemical and biophysical research communications* **454**, 137-143 (2014).
12. Golledge, J. Abdominal aortic aneurysm: update on pathogenesis and medical treatments. *Nat Rev Cardiol* **16**, 225-242 (2019).
13. Bartoli, M.A., *et al.* In vivo assessment of murine elastase-induced abdominal aortic aneurysm with high resolution magnetic resonance imaging. *European journal of vascular and endovascular surgery : the official journal of the European Society for Vascular Surgery* **44**, 475-481 (2012).
14. Koulaxouzidis, G., Reim, G. & Witzel, C. Fibrin glue repair leads to enhanced axonal elongation during early peripheral nerve regeneration in an in vivo mouse model. *Neural Regen Res* **10**, 1166-1171 (2015).
15. Menovsky, T. & Beek, J.F. Laser, fibrin glue, or suture repair of peripheral nerves: a comparative functional, histological, and morphometric study in the rat sciatic nerve. *J Neurosurg* **95**, 694-699 (2001).

Supplementary Information for

The Self-healing Polyurethane-elastomer with Mechanical Tunability for Multiple Biomedical Applications in vivo

Chenyu Jiang, Luzhi Zhang, Qi Yang, Shixing Huang, Hongpeng Shi, Qiang Long, Bei Qian, Qiang Zhao*, Zhengwei You*, Xiaofeng Ye*

***Corresponding author**:

Email: zq11607@rjh.com.cn; zyou@dhu.edu.cn; xiaofengye@hotmail.com

Supplementary Figures

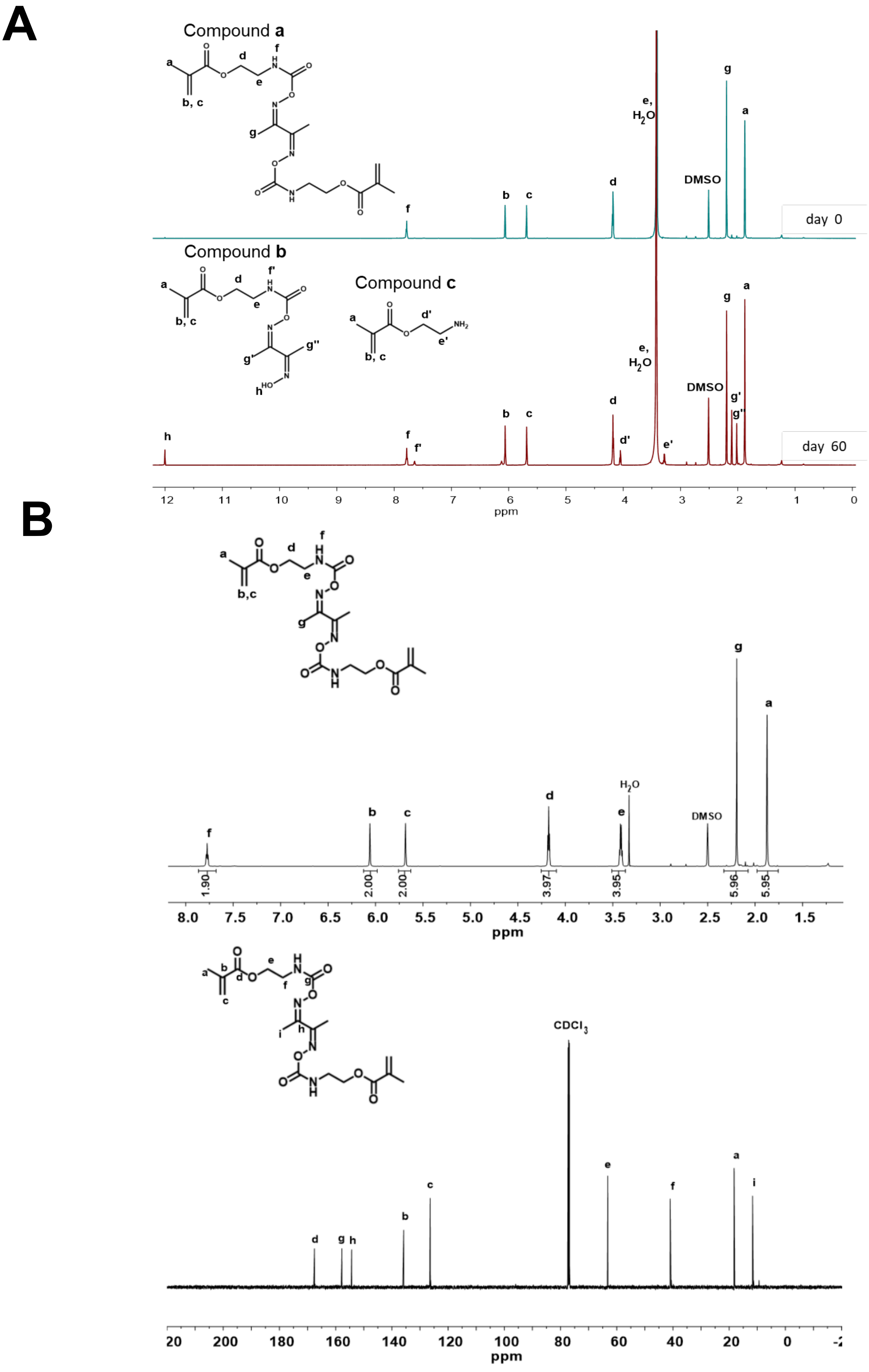

Supplementary Figure 1. The ^{1}H NMR spectra of the mixture of compound a and water at day 0 and day 60 and ^{1}H NMR and ^{13}C NMR spectra of compound a

(A) Compound **a** and water were mixed and dissolved in DMSO-d6. NMR spectra were used to monitor the reaction. After 60 days, compounds **b** and **c** were formed due to the hydrolysis of the oxime-urethane bond. It was calculated that approximately 21% of the oxime-urethane bonds were hydrolyzed. (B) ^{1}H NMR (top) and ^{13}C NMR (bottom) spectra of compound **a**

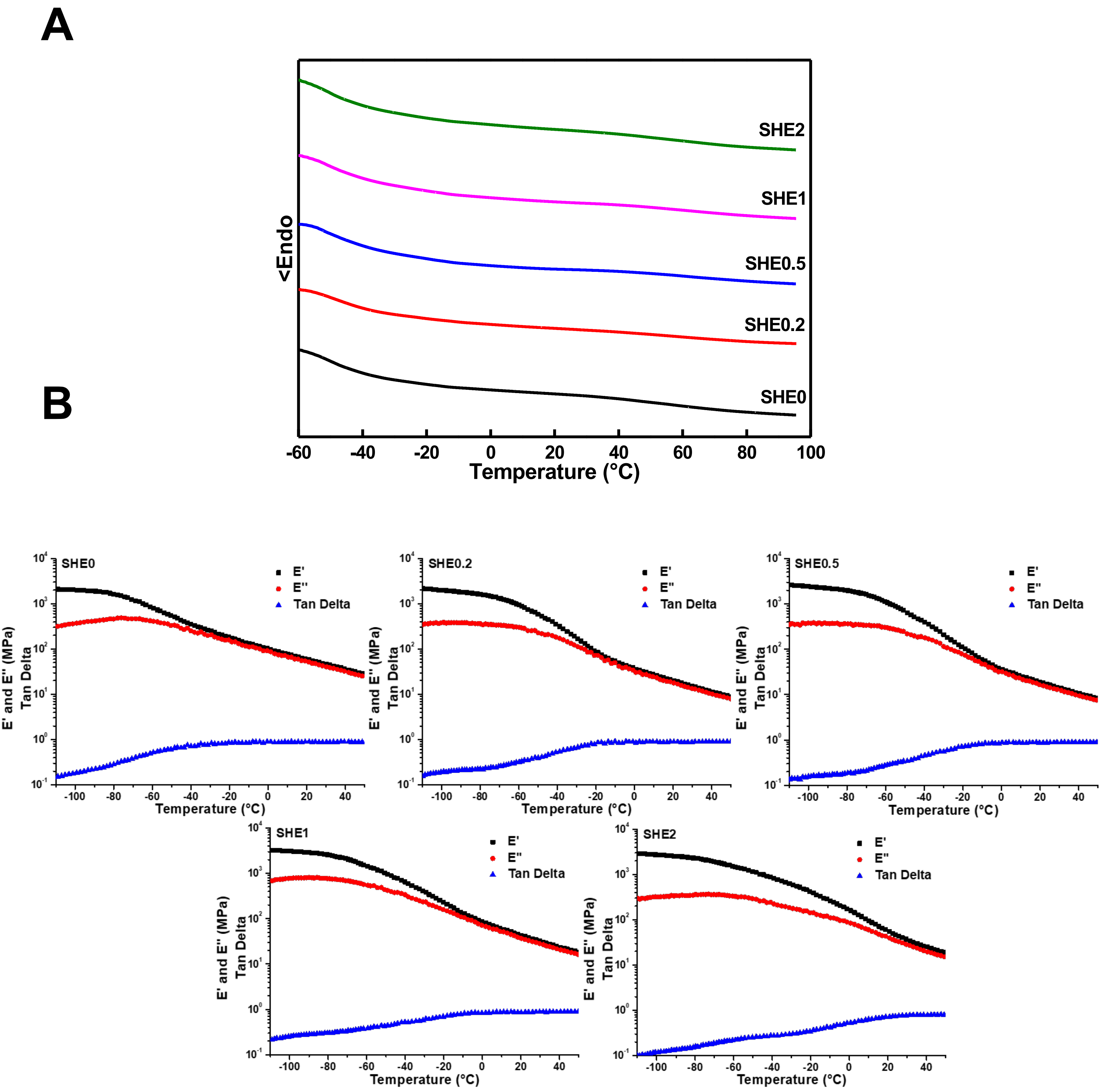

Supplementary Figure 2. DSC curves and DMA test of the SHEs.

(A)DSC were performed on a TA-Q20 differential scanning calorimeter. Samples were heated from 25℃ to 100 ℃, cooled to -80 ℃, and reheated to 100 ℃ at a rate of 10 ℃ $\cdot min^{-1}$ under a nitrogen atmosphere. The DSC curves of SHEs did not show glass transition, crystallization or melting peaks from -60 to 100 ℃. (B) DMA tests revealed the glass transition temperature of the self-healing elastomers. The glass transition temperature of SHE0, SHE0.2, SHE0.5, SHE1, SHE2 were -35.7℃, -21.2℃, -18.1℃, -7.2℃ and 20.6℃, respectively.

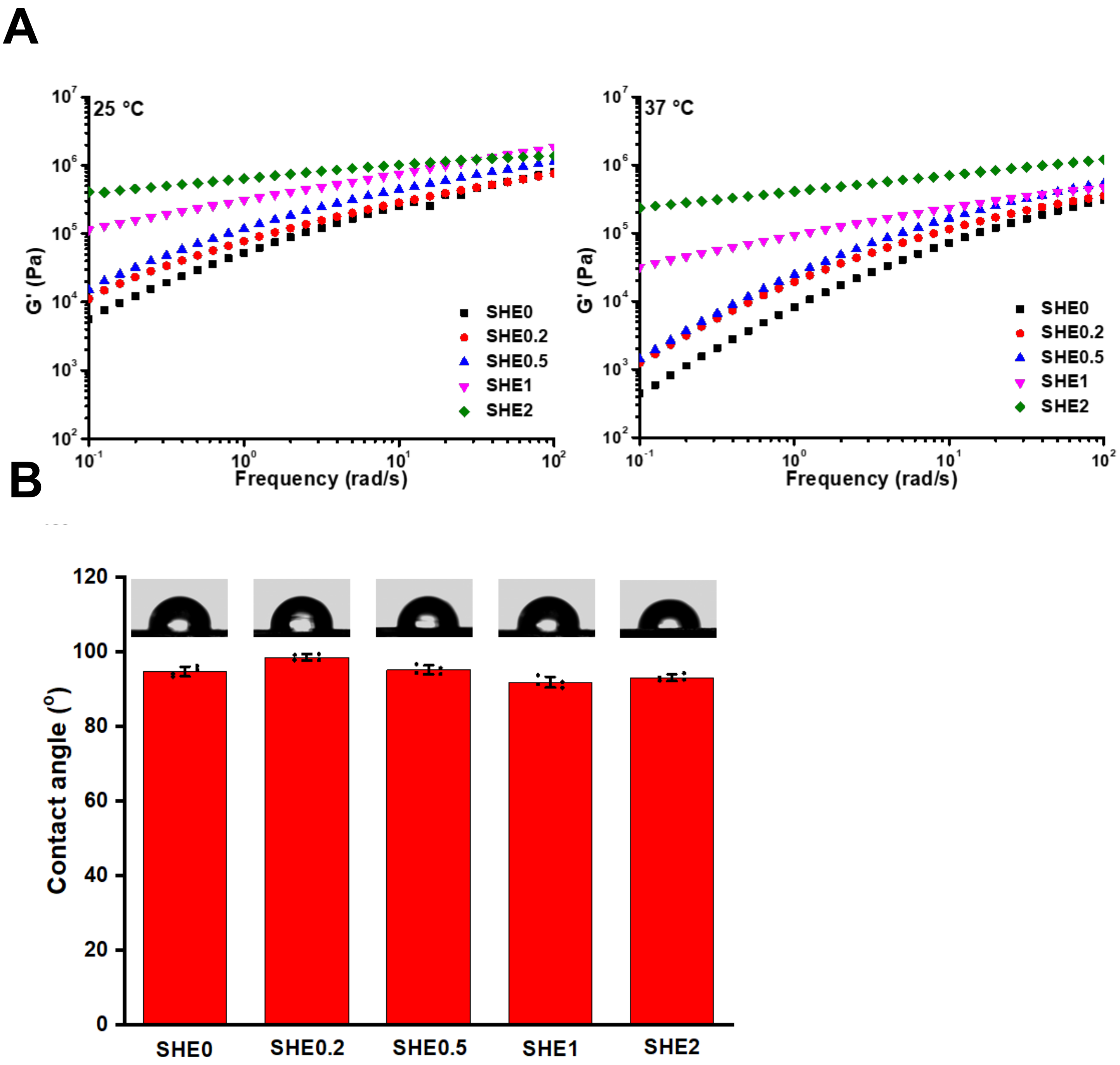

Supplementary Figure 3. Rheological test and contact angle test of SHEs. (A) According to the rheological test, addition of the crosslinking points to SHEs were observed to enhance the mechanical strength of the polymer at both 25 °C and 37 °C. (B) Water contact angle measurements were performed to assess the response of the material to aqueous environments. The contact angles (92° — 99°) revealed the hydrophobicity of the materials (n =4 in each group). Data were presented as mean ± s.d.

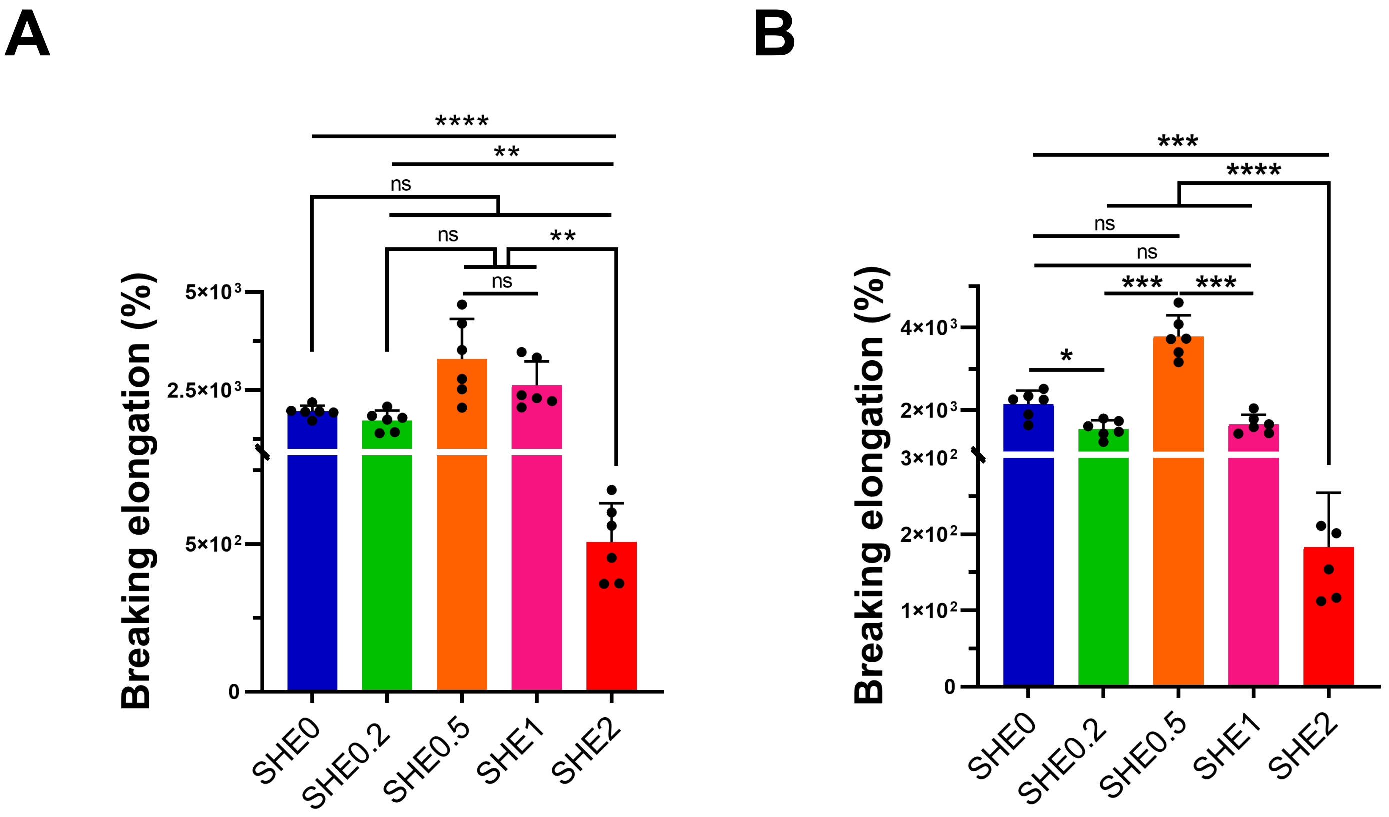

Supplementary Figure 4. Breaking elongation of SHEs before and after self-healing.

(A) Histogram shows the breaking elongation of SHEs before self-healing (n = 6 in each group). (B) Histogram shows the breaking elongation of SHEs after self-healing (n = 6 in each group). Data were presented as mean ± s.d. Brown-Forsythe ANOVA test with Dunnett's multiple comparison test (A, B) was used for comparing tensile strength and elastic modulus in groups before and after self-healing. Breaking elongation before self-healing (A): SHE0.2, SHE0.5 and SHE1 compared to SHE0, ns p = 0.4985, 0.1466, 0.2415 respectively; SHE0 compared to SHE2, ****p < 0.0001; SHE0.5 and SHE1 compared to SHE0.2, ns p = 0.0719 and 0.0882 respectively; SHE0.2 compared to SHE2, ***p = 0.0002; SHE0.5 compared to SHE1, ns p = 0.8121; SHE0.5 and SHE1 compared to SHE2, **p = 0.008 and 0.0029 respectively. Breaking elongation before self-healing (B): SHE0 compared to SHE0.2, *p = 0.0322; SHE0 compared to SHE0.5, **p = 0.0014; SHE0 compared to SHE1, ns p = 0.1308; SHE0 compared to SHE2, ***p = 0.0002; SHE0.2 compared to SHE0.5, ***p = 0.0002; SHE0.2 compared to SHE1, ns p = 0.9848; SHE0.2, SHE0.5 and SHE1 compared to SHE2, ****p < 0.0001; SHE0.5 compared to SHE1, ***p = 0.0003. Source data are provided as a Source Data file.

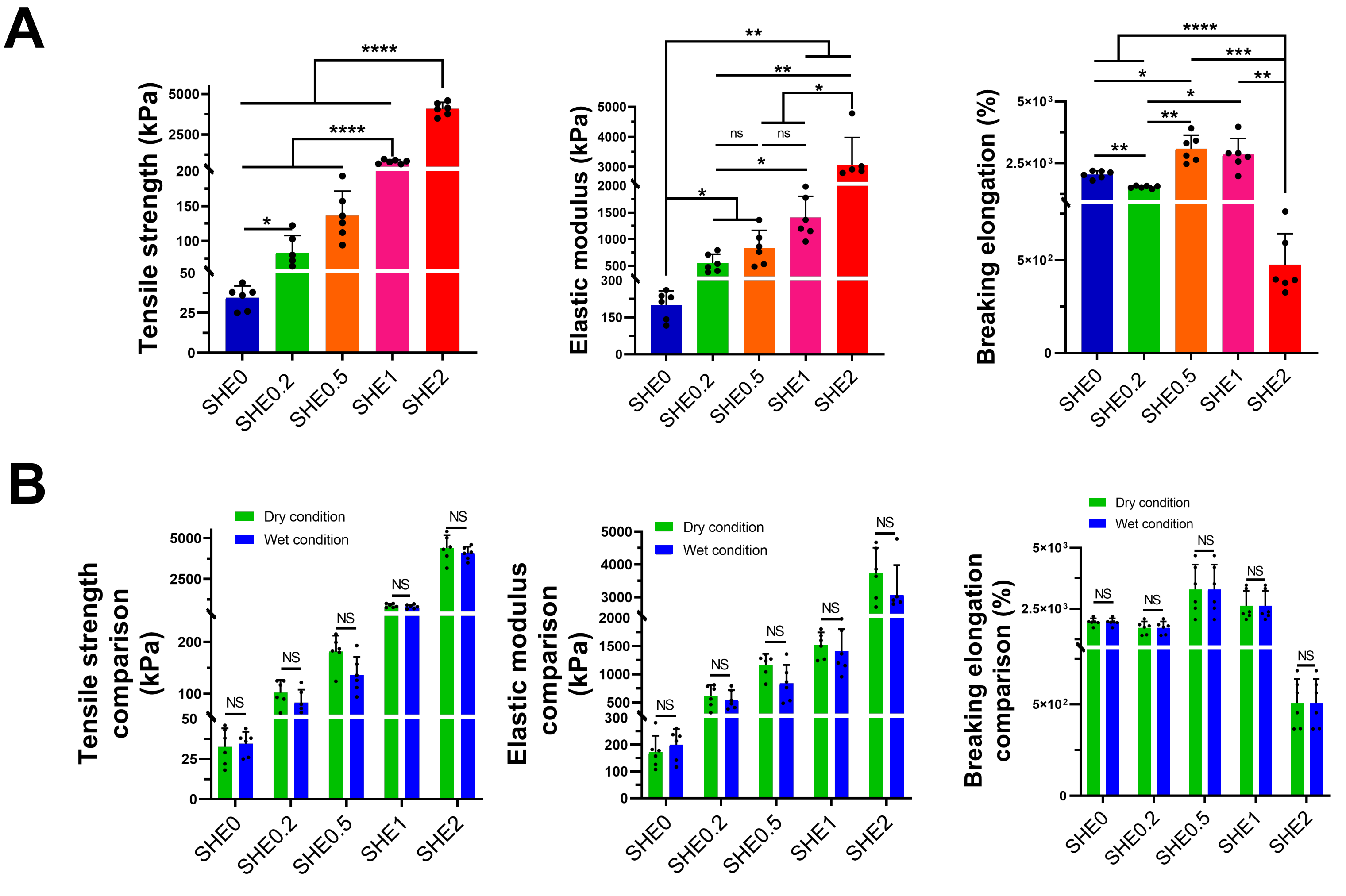

Supplementary Figure 5. Mechanical properties of SHEs in wet condition

To analyze the mechanical properties of SHEs in wet condition, SHEs were immersed in saline for 2 h to mimic wet physiological condition before tensile test. (A) Histograms show the tensile strength, elastic modulus and breaking elongation of SHEs in wet condition (n=6 in each group). (B) Histograms show the comparison of tensile strength, elastic modulus and breaking elongation of SHEs between dry and wet condition (n=6 in each group). Data were presented as mean ± s.d. Brown-Forsythe ANOVA test with Dunnett's multiple comparison test (A) was used for comparing tensile strength, elastic modulus and breaking elongation in wet condition. Tensile strength in wet conditon (A left): SHE0, SHE0.2, SHE0.5 and SHE1 compared to SHE2, ****p < 0.0001; SHE0, SHE0.2 and SHE0.5 compared to SHE1, ****p < 0.0001; SHE0 compared to SHE0.2, *p = 0.0275; Elastic modulus in wet condition (A middle): SHE0.2 and SHE0.5 compared to SHE0, *p = 0.207 and 0.035 respectively; SHE1 and SHE2 compared to SHE0, **p = 0.0051 and 0.0043 respectively; SHE0.2 and SHE1 compared to SHE0.5, ns p = 0.5089 and 0.1662 respectively; SHE0.2 compared to SHE1, *p = 0.0145; SHE0.2 compared to SHE2, **p = 0.0084; SHE0.5 and SHE1 compared to SHE2, *p = 0.0104 and 0.0365 respectively. Breaking elongation in wet condition (A right): SHE0 and SHE0.2 compared to SHE2, ****p < 0.0001; SHE0 compared to SHE0.2, **p = 0.002; SHE0 compared to SHE0.5, *p = 0.0289; SHE0.2 compared to SHE0.5, **p = 0.0066; SHE0.2 compared to SHE1, *p = 0.0293; SHE0.5 compared to SHE2, ***p = 0.0002; SHE1 compared to SHE2, **p = 0.001. Two tailed unpaired t test (B) was used for comparing the differences of tensile strength, elastic modulus and breaking elongation between dry condition and wet condition in one certain SHE. Tensile strength comparison (B left): of SHE0, ns p > 0.9999; of SHE0.2, ns p > 0.9999; of SHE0.5, ns p = 0.996; of SHE1, ns p = 0.9991; of SHE2, ns p = 0.3242. Elastic modulus comparison (B middle): of SHE0, ns p > 0.9999; of SHE0.2, ns p = 0.9998; of SHE0.5, ns p = 0.6665; of SHE1, ns p = 0.9952; of SHE2, ns p = 0.0537. Breaking elongation comparison (B right): of SHE0, ns p > 0.9999; of SHE0.2, ns p > 0.9999; of SHE0.5, ns p > 0.9999; of SHE1, ns p > 0.9999; of SHE2, ns p > 0.9999. Source data are provided as a Source Data file.

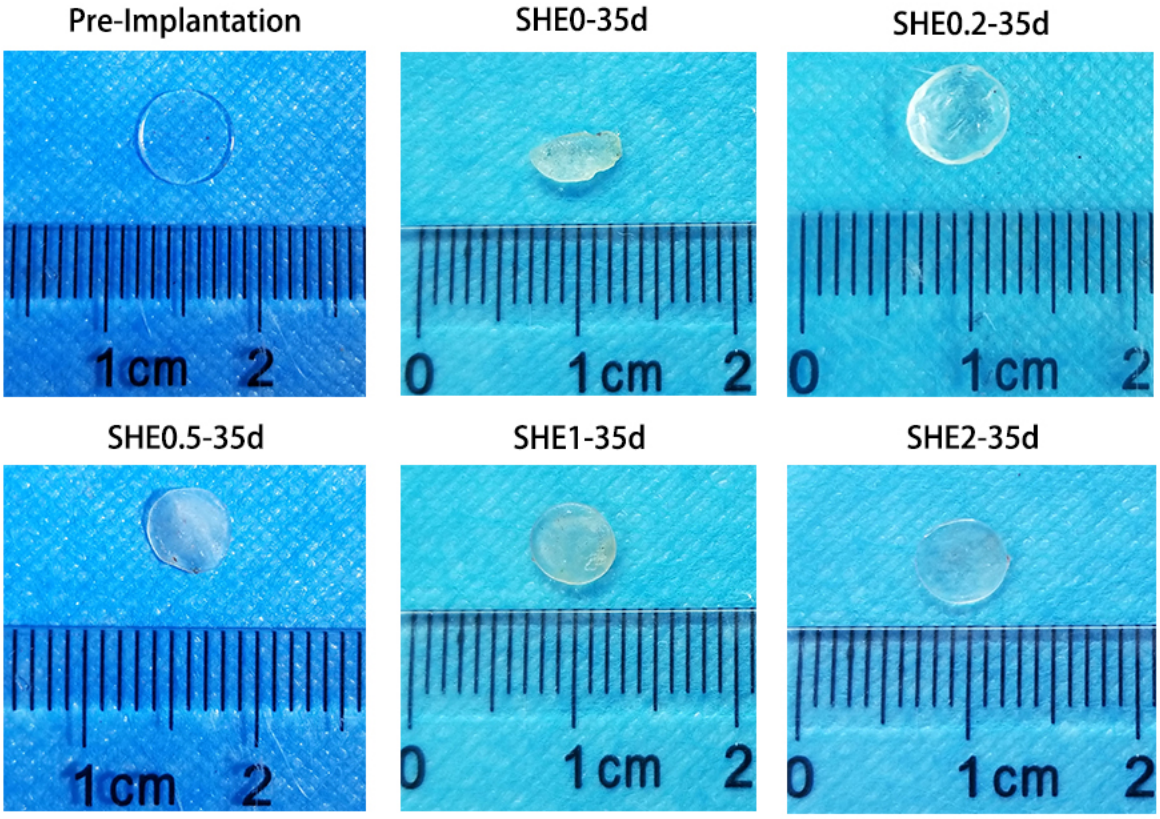

Supplementary Figure 6. Photos of SHEs of pre-implantation and post-implantation.

Physical pictures on day 35 of subcutaneous implantation. The crosslinked SHEs(0.2-2) maintained their original shape after 35 days implantation.

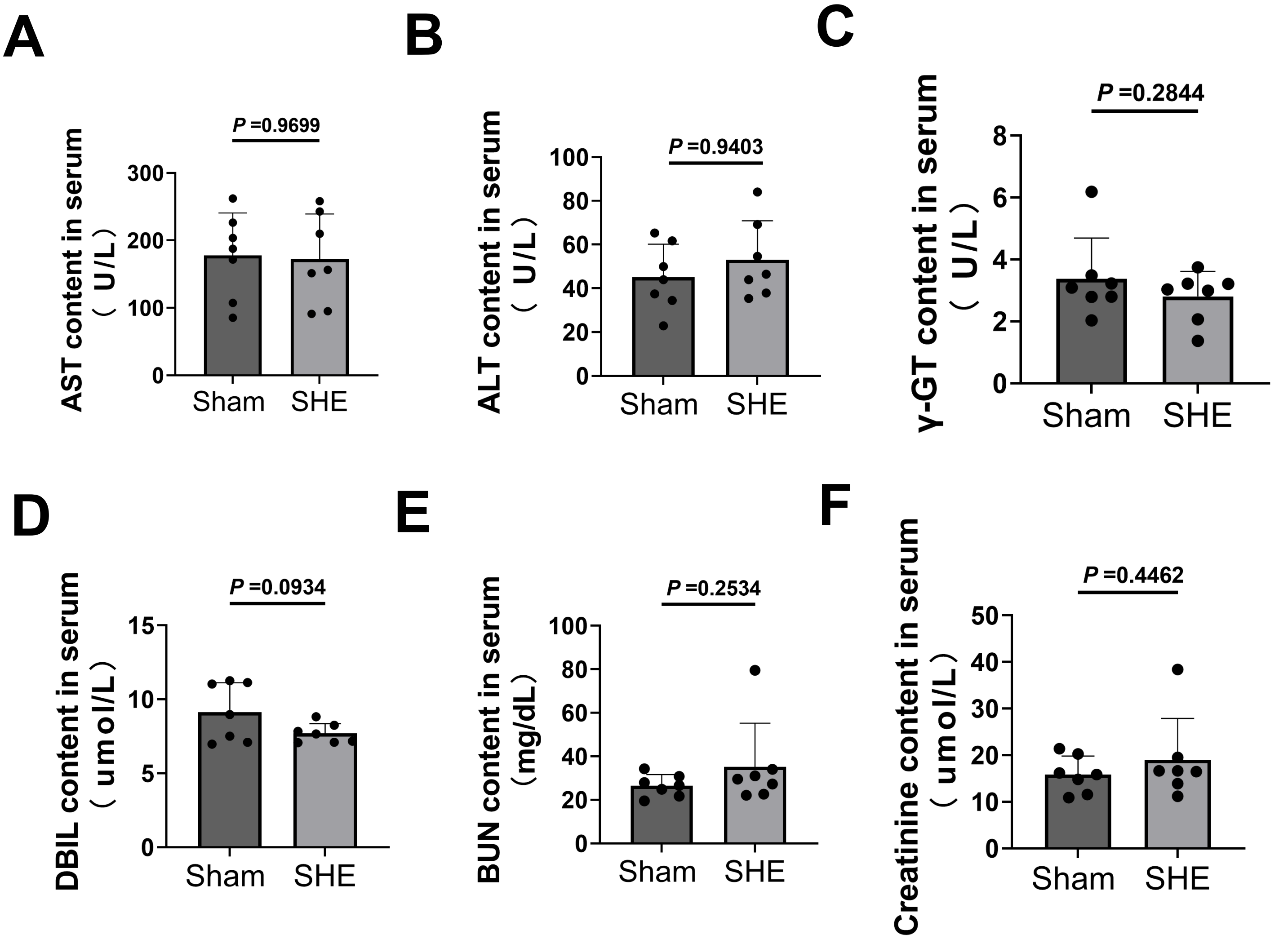

Supplementary Figure 7. Liver and renal function analysis of SHE *in vivo*. Serum of animals were extracted on the day 35 post of elastomer implantation. (A and B) Liver function indicators including alanine aminotransferase (ALT), aspartate aminotransferase (AST), the histograms showed no statistical difference of three indicators in each group (n = 5 in each group). (C and D) γ-glutamyl transpeptidase (γ-GT) and direct bilirubin (DBIL) were tested in sham and SHE group. The histograms showed no statistical difference of three indicators in each group. (E and F)) Renal function indicators including blood urea nitrogen (BUN) and serum creatinine (SCr) were tested in sham and SHE groups. The histograms showed no statistical difference of two indicators in each group (n = 5 in each group) . Data were presented as mean ± s.d. Two tailed unpaired t test (A through F) was used for comparing the difference of hepatic function and renal function in sham and SHE group. Source data are provided as a Source Data file. AST, aspartate aminotransferase; ALT, alanine aminotransferase; γ-GT, γ-glutamyl transpeptidase; DBIL, direct bilirubin; BUN, urea nitrogen.

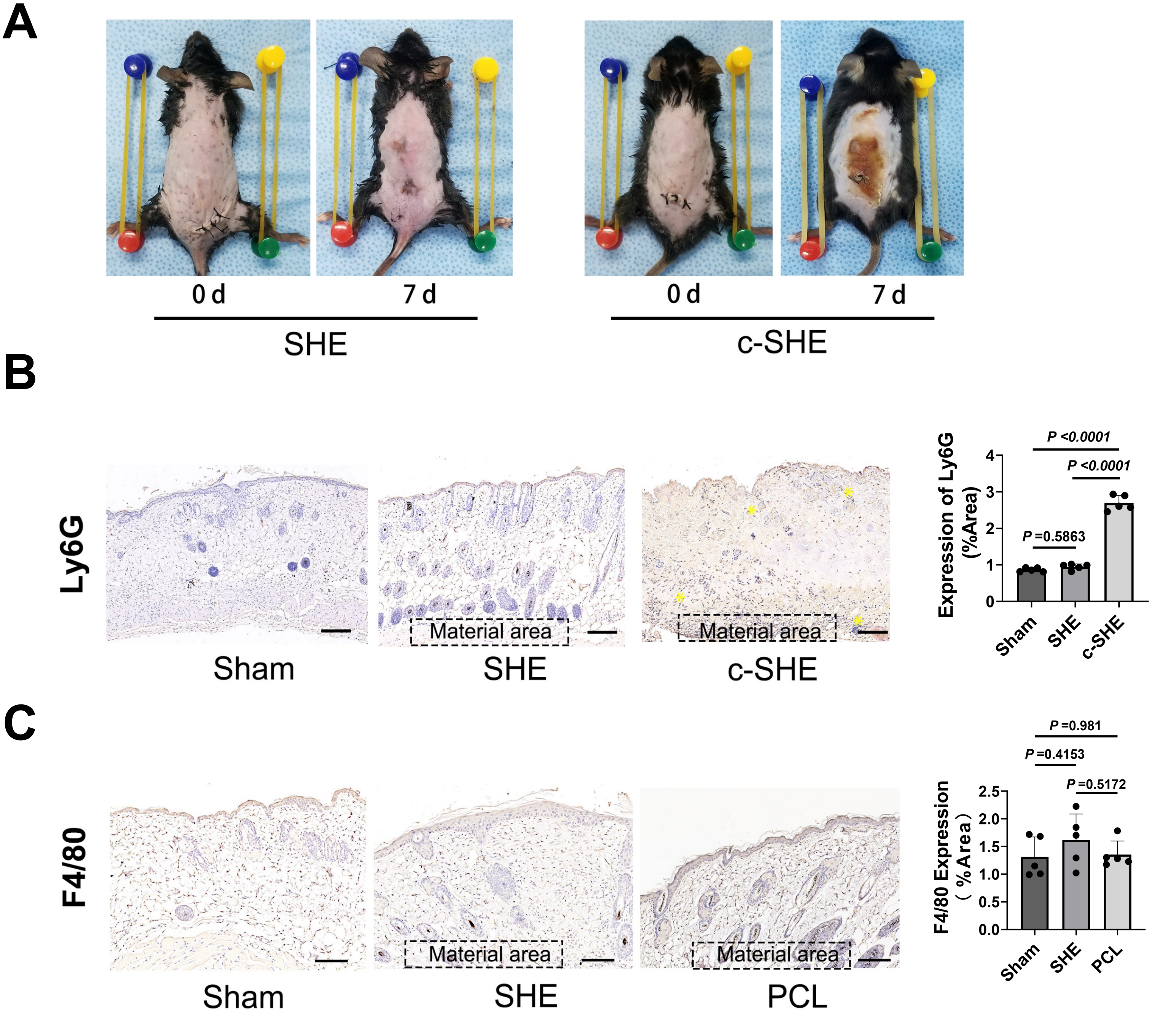

Supplementary Figure 8. Subcutaneous inflammatory response.

(A) The back skin of mouse was intact without inflammatory after instantaneous implantation. On the day 7 post of implantation, the skin of back implanted with c-SHE showed large-scale inflammation with the presence of dark orange skin ulceration while only presence of surgical scar was observed on back of mouse implanted with SHE. (B) Immunohistochemical staining of Ly6G (a marker for mouse neutrophils as a acute inflammation indicator) for analyzing the acute subcutaneous inflammation response. SHE showed intact skin structure without inflammatory cell infiltration while c-SHE group showed that the structure of the skin was destroyed. The structure of the hair follicles disappeared, and a large number of inflammatory cells infiltrated. Positive staining areas were marked by yellow*. Statistical histogram of Ly6G expression at day 7 showed statistical difference between SHE group (using SHE2) and c-SHE group (as a negative control) ($n = 5$ in each group). (C) Immunohistochemical staining of F4/80 (a marker for mouse macrophage) for analyzing the chronic subcutaneous inflammation response in sham, SHE (using SHE2) and PCL (as a positive control) groups. There was no major difference of skin structure among sham, SHE and PCL group. Statistical histogram of F4/80 expression at day 14 shows no difference among sham, SHE and PCL groups ($n = 5$ in each group). Scale bar = 100 μm. Data were presented as mean ± s.d. Ordinary one-way ANOVA test with Tukey's multiple comparisons test (B, C) was used for comparing the difference of skin inflammation in groups. Expression of Ly6G (B): Sham compared to SHE, ns $p = 0.5863$; Sham compared to c-SHE, ****$p < 0.0001$; SHE compared to c-SHE, ****$p < 0.0001$. Expression of F4/80 (C): Sham compared to SHE, ns $p = 0.4153$; Sham compared to PCL, ns $p = 0.981$; SHE compared to PCL, ns $p = 0.5172$. Source data are provided as a Source Data file. SHE, Self-healing elastomer; PCL, polycaprolactone; c-SHE, containing catalyst self-healing elastomer.

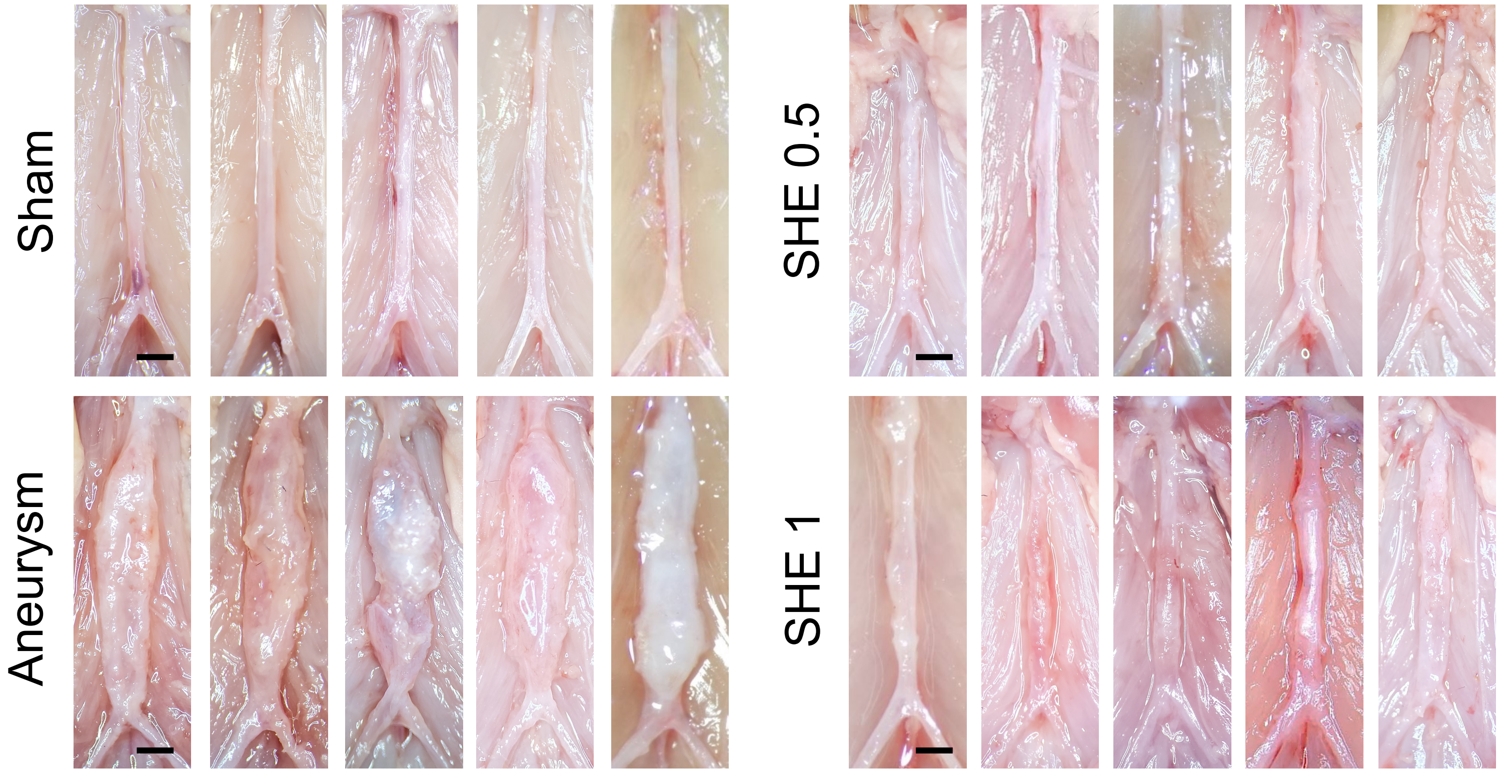

Supplementary Figure 9. Morphometry picture of aorta in sham, aneurysm, SHE0.5 and SHE 1 group.
Scale bar = 1mm.

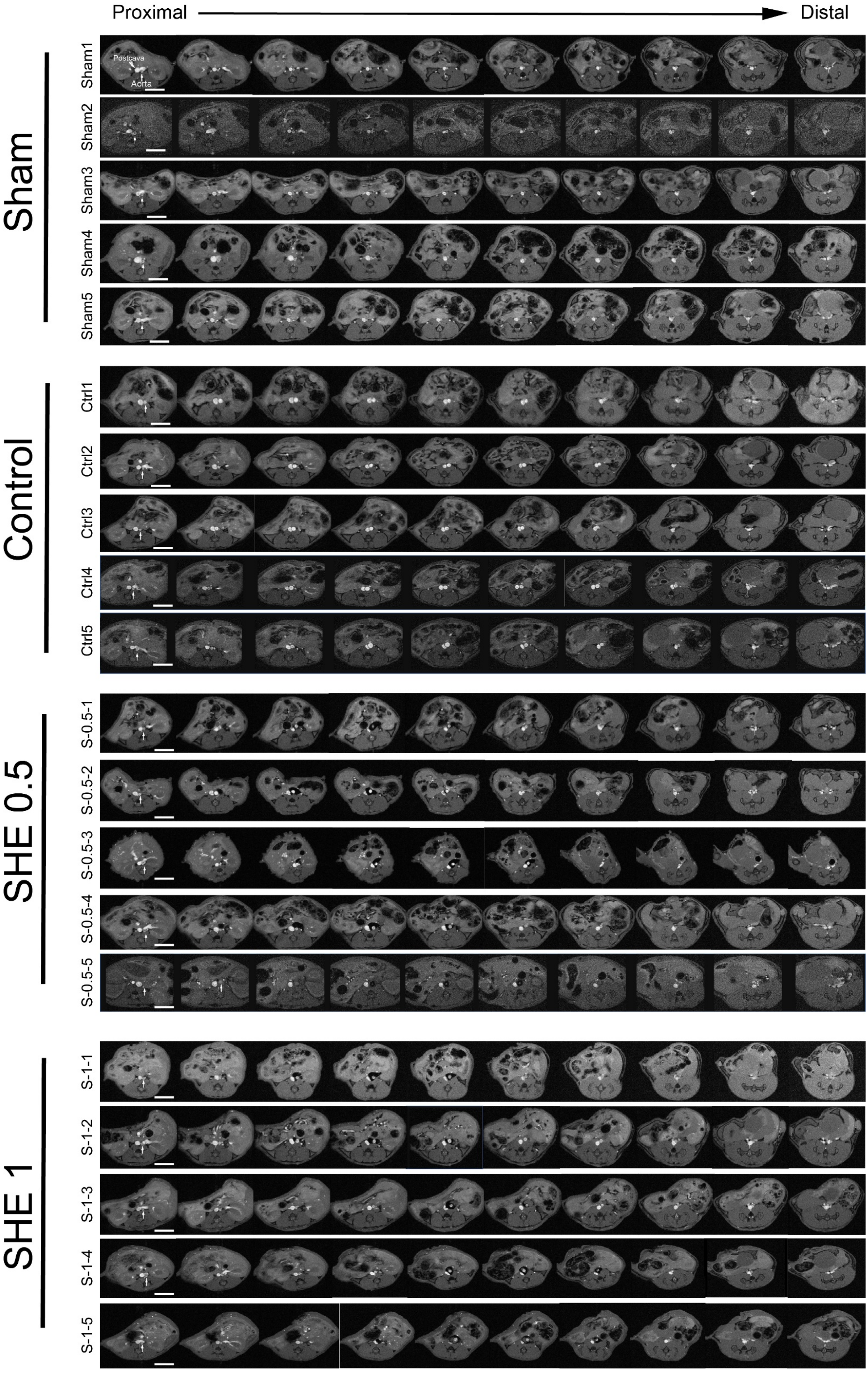

Supplementary Figure 10. MRI imaging summary of aorta in the in sham, aneurysm, SHE0.5 and SHE1 group.
The vessel marked with white arrow is abdominal aorta.
Scale bar = 5mm.

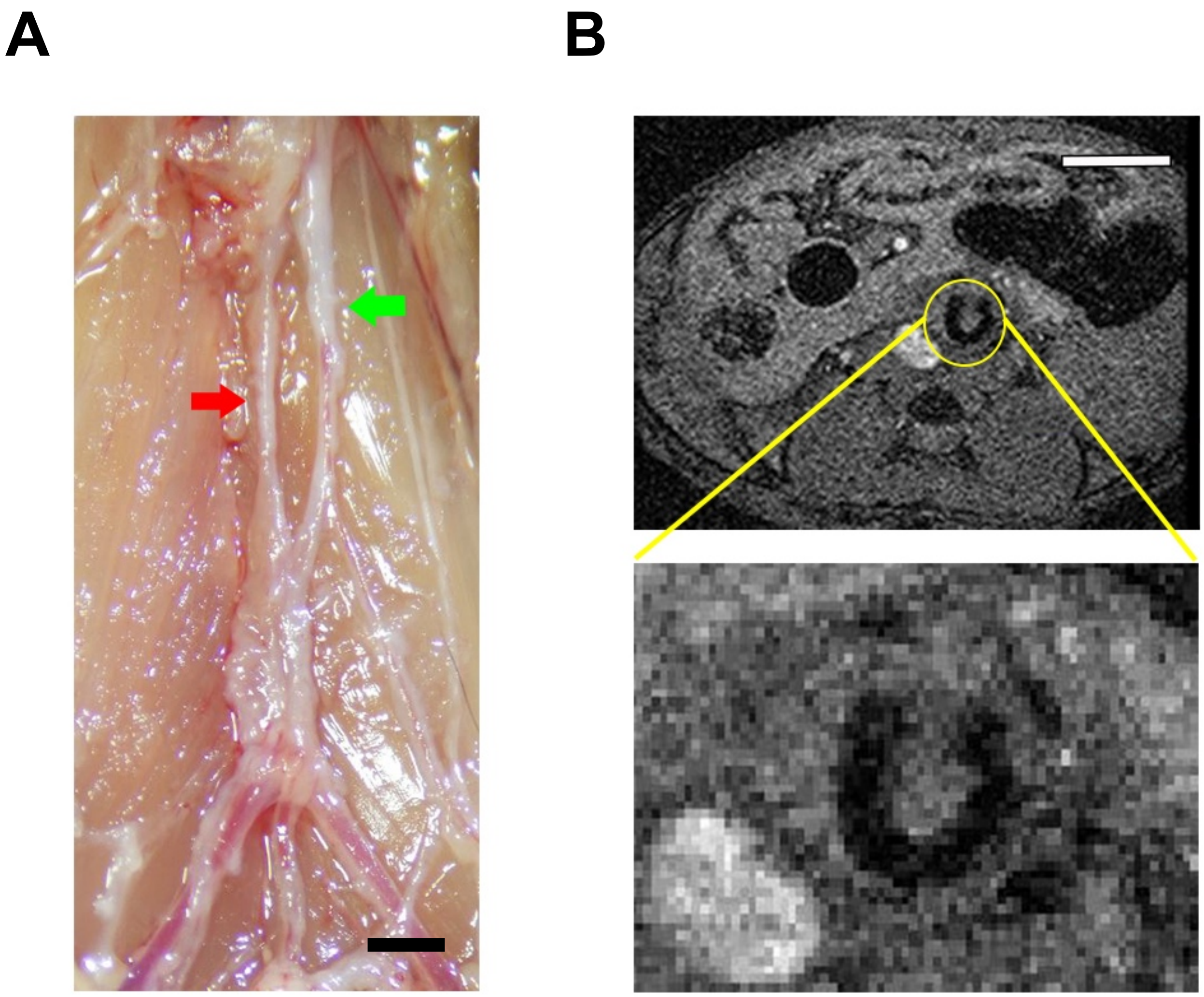

Supplementary Figure 11. Gross observation and MRI imagination of SHE2 wrapped aneurysm.

(A)Aneurysm at 30x magnification, red arrow shows the SHE2 induced injury of aneurysm (marked with red arrow) which was blocked by thrombus, green arrow shows the collateral circulation maintaining the normal circulation. Scale bar = 1mm (B) The MRI image inside a solid yellow circle shows no flowing void effect in vessel meaning a occlusion in the lumen of a damaged vessel. Scale bar = 5mm.

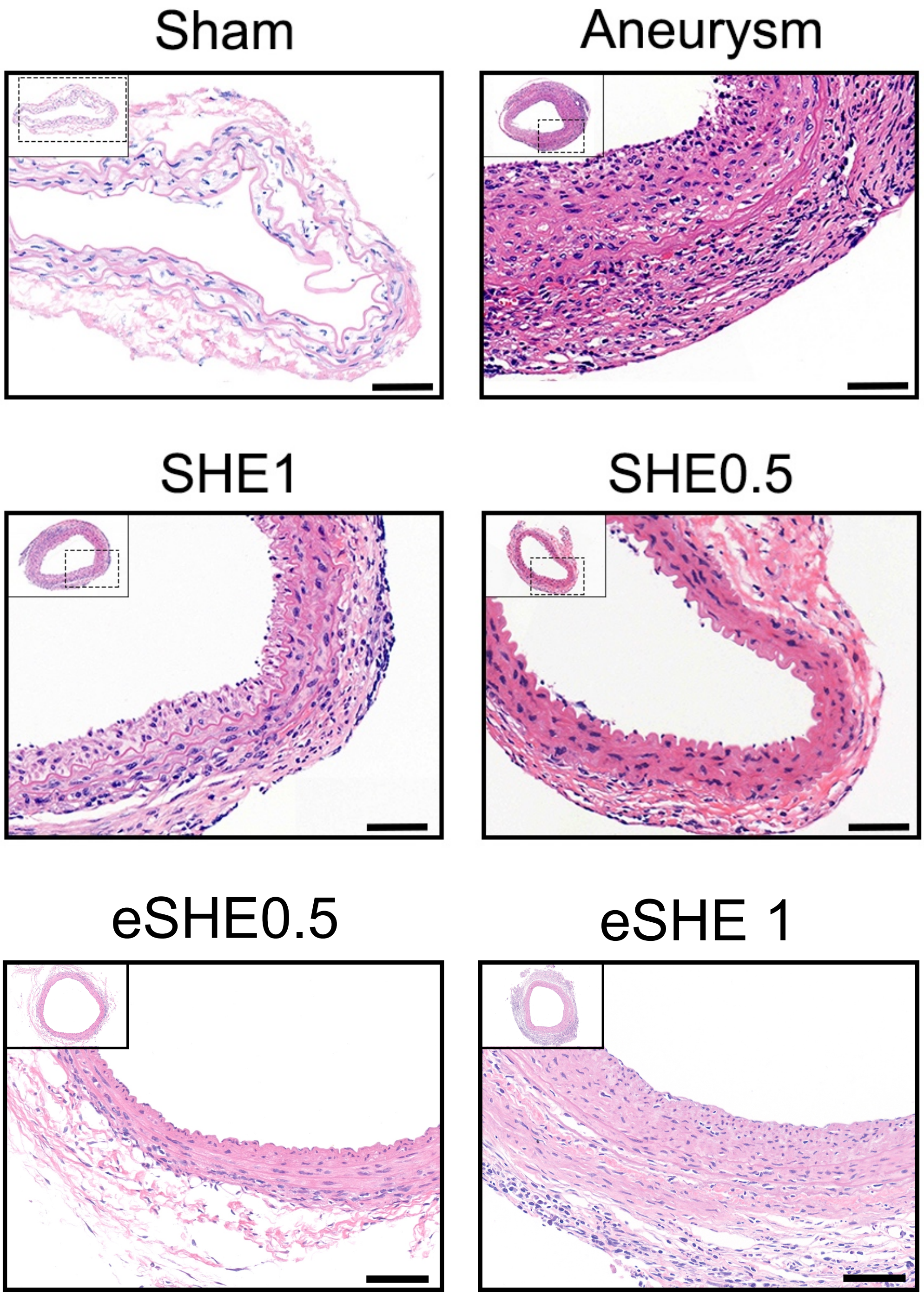

Supplementary Figure 12. HE staining of aorta in transverse section. Transverse sections of aorta were stained with hematoxylin-eosin in sham, aneurysm, SHE1, SHE0.5, eSHE0.5 and eSHE1 groups, each experiment was repeated three times . Compared with the sham group, the remaining five groups all had thickened blood vessel walls to varying degrees, and the number of nucleated cells increased. Scale bar = 50 μm.

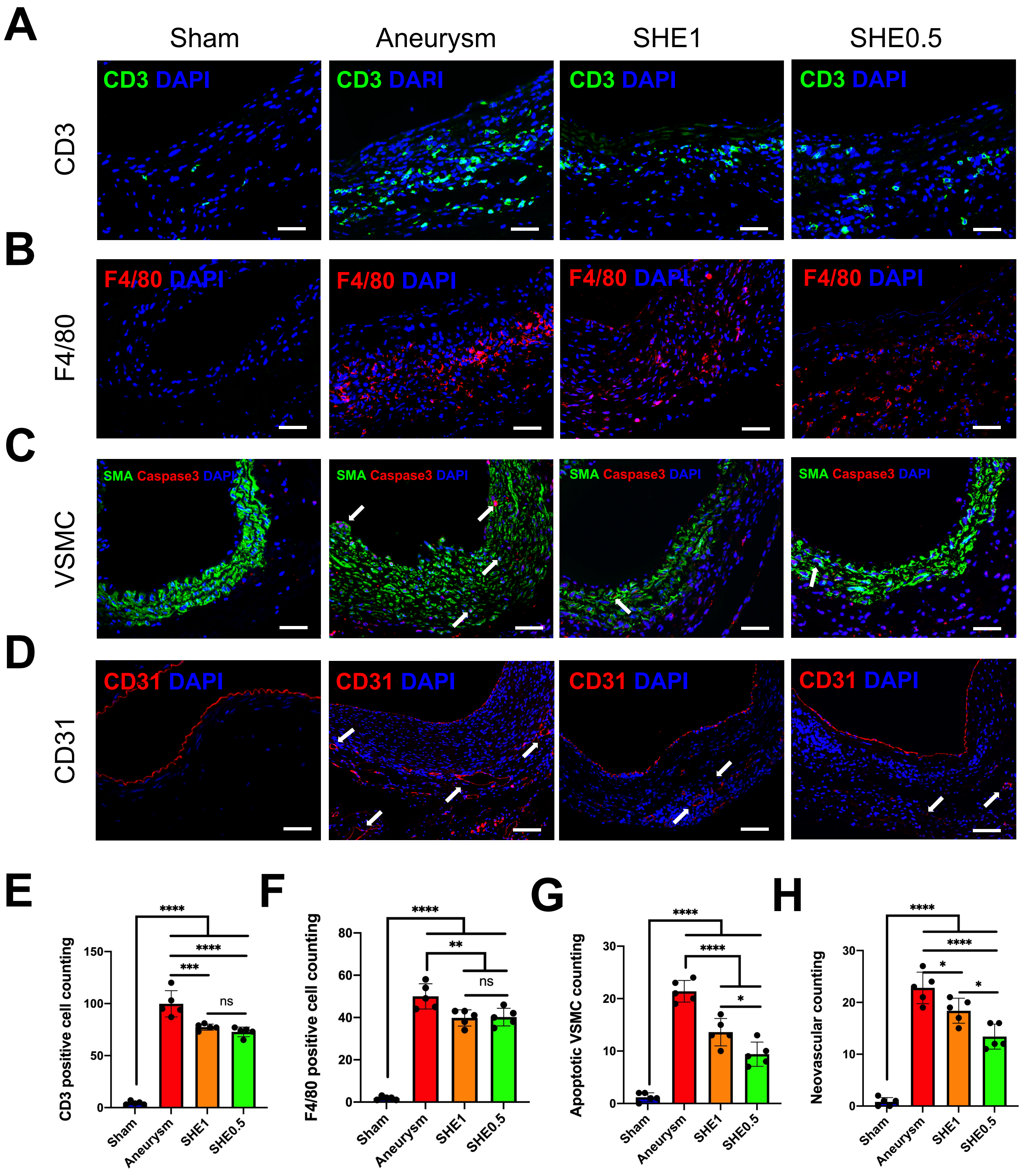
A
Sham
Aneurysm
SHE1
SHE0.5
CD3
CD3 DAPI
B
F4/80
F4/80 DAPI
C
VSMC
SMA Caspase3 DAPI
D
CD31
CD31 DAPI
E
CD3 positive cell counting
****
***
ns
F
F4/80 positive cell counting
**
G
Apoptotic VSMC counting
*
H
Neovascular counting
Sham
Aneurysm
SHE1
SHE0.5

Supplementary Figure 13. Inflammation, apoptosis of vascular smooth muscle cell and neovascularization of aneurysm after SHE wrapping.

(A and B) Transverse sections of aorta were stained with CD3 and F4/80 to illustrate lymphocyte and macrophage infiltration in each group. Green marked lymphocyte and red marked macrophage. Scale bar = 40μm. (C) Dual labeling of α-SMA and caspase-3 in each group. Green marked vascular smooth muscle cell and red marked caspase-3. Scale bar = 40μm. (D) CD31 labeling to illustrate neovascular. The neovascular is marked with white arrow. Scale bar = 40μm. (E through H) The statistic histogram of counting of lymphocyte, macrophage, apoptotic VSMC and neovascular respectively per high power filed (n = 5 in each group). Data were presented as mean ± s.d. Ordinary one-way ANOVA test with Tukey's multiple comparisons test (E, F, G, H) was used for comparing the difference of CD3 cell counting, F4/80 positive cell counting, apoptosis VSMC counting and neovascular counting in groups. CD3 positive cell counting (E): Aneurysm, SHE1 and SHE0.5 group compared to SHE0 group, ****p < 0.0001; Aneurysm compared to SHE1, ***p = 0.0006; Aneurysm compared to SHE0.5, ****p < 0.0001; SHE1 compared to SHE0.5, ns p = 0.7243. F4/80 positive cell counting (F): Aneurysm, SHE1 and SHE0.5 group compared to Sham group, ****p < 0.0001; SHE1 and SHE0.5 group compared to Aneurysm group, **p = 0.0062 and 0.0084 respectively; SHE1 compared to SHE0.5, ns p = 0.9987. Apoptotic VSMC counting (G): Aneurysm, SHE1 and SHE0.5 group compared to SHE0 group, ****p < 0.0001; SHE1 and SHE0.5 group compared to Aneurysm group, ****p < 0.0001; SHE1 group compared to SHE0.5 group, *p = 0.0252. Neovessel counting (H): Aneurysm, SHE1 and SHE0.5 group compared to SHE0 group, ****p < 0.0001; Aneurysm and SHE0.5 group compared to SHE1 group, *p = 0.0382 and 0.017 respectively; Aneurysm group compared to SHE0.5 group, ****p < 0.0001. Source data are provided as a Source Data file. VSMC, vascular smooth muscle cell.

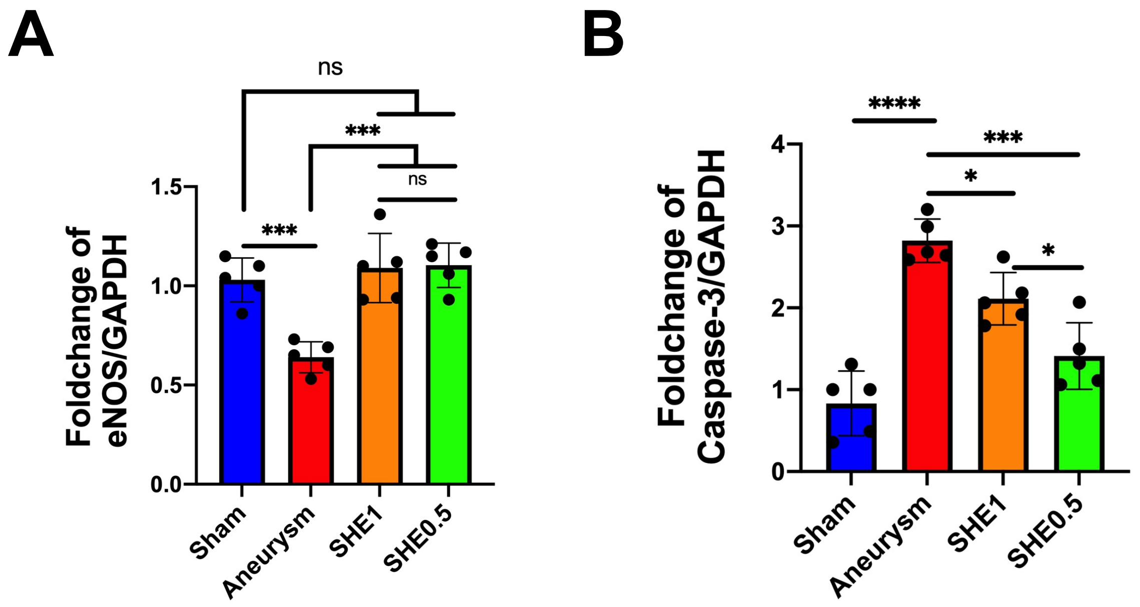

Supplementary Figure 14. mRNA transcriptional changes of eNOS and caspase-3.

(A) The statistical histogram of eNOS mRNA transcriptional changes in sham, aneurysm, SHE1 and SHE0.5 group (n = 5 in each group). GAPDH was used as internal reference. (B) The statistical histogram of caspase-3 mRNA transcriptional changes in sham, aneurysm, SHE1 and SHE0.5 group (n = 5 in each group). GAPDH was used as internal reference. Data were presented as mean ± s.d. Ordinary one-way ANOVA test with Tukey's multiple comparisons test (A, B) was used for comparing the difference of eNOS transcription foldchange and caspase-3 transcription foldchange in groups. Foldchange of eNOS transcription (A): Aneurysm group compared to sham group, ***p = 0.0007; SHE1 and SHE0.5 group compared to Sham group, ns p = 0.8686 and 0.7817 respectively; SHE1 and SHE0.5 group compared to Aneurysm group, ***p = 0.0002 and 0.0001 respectively; SHE1group compared to SHE0.5 group, ns p = 0.9979. Foldchange of caspase-3 transcription (B): Sham group compared to Aneurysm group, ****p < 0.0001; Sham group compared to SHE1 group, ***p = 0.0002; Aneurysm and SHE0.5 group compared to SHE1, *p = 0.0267 and 0.0287 respectively; Aneurysm group compared to SHE0.5 group, ****p < 0.0001. Source data are provided as a Source Data file. GADPH, glyceraldehyde 3-phosphate dehydrogenase.

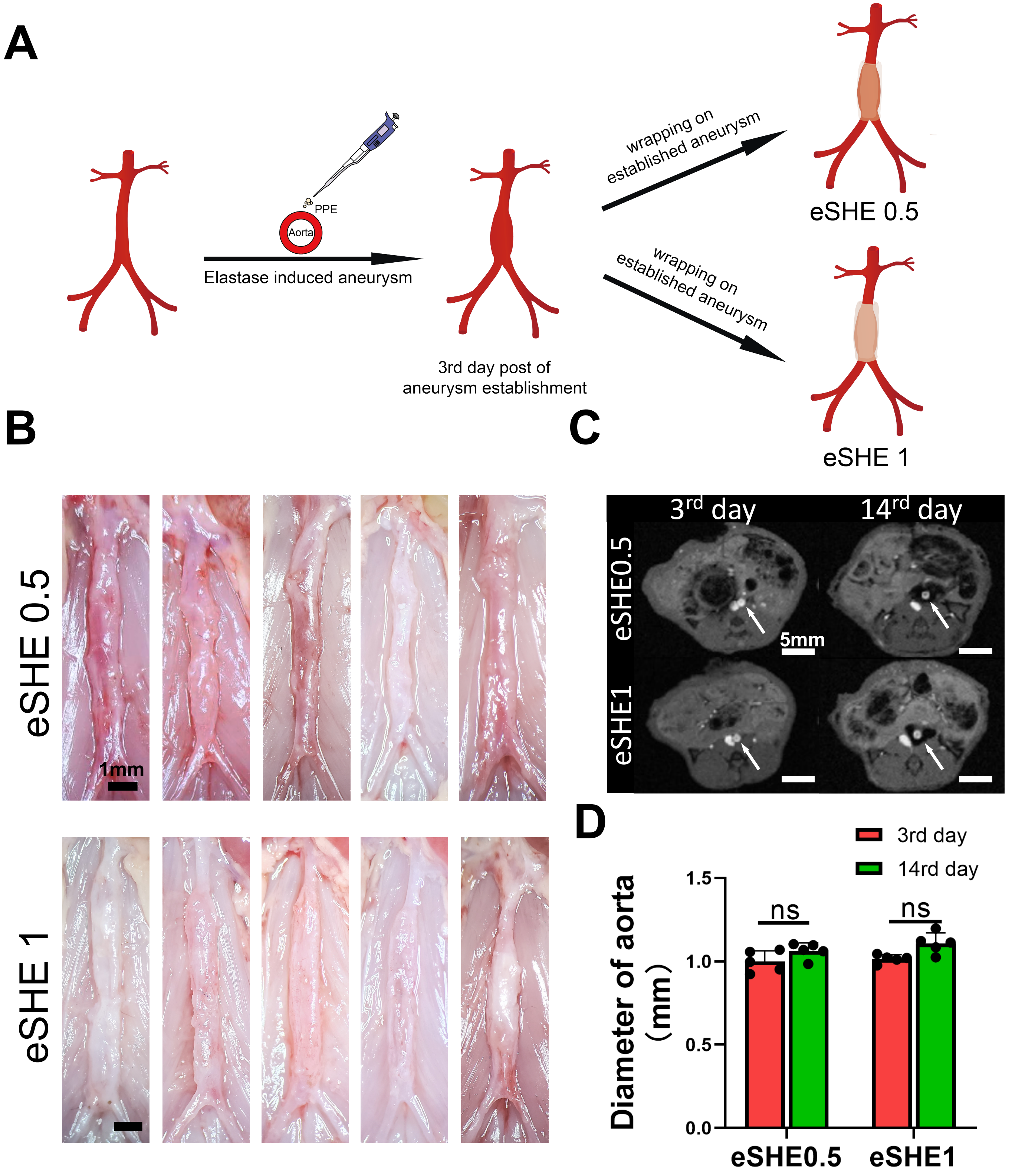

Supplementary Figure 15. Pattern, morphometry and MRI picture of aorta in eSHE0.5 and eSHE 1 group.

(A) Pattern diagram of SHE wrapping on 3rd-day established aneurysm. PPE: Porcine pancreatic elastase (B) Morphometry picture of abdominal aorta on 14rd day post of aneurysm establish in the eSHE0.5 and eSHE1 group. Scale bar = 1mm. (C) MRI picture of abdominal aorta on 3rd day post of aneurysm establish and 14rd day post of aneurysm establish respectively in the eSHE0.5 and eSHE1 group. The vessel marked with white arrow is abdominal aorta. Scale bar = 5mm. (D) The statistic histogram of aorta diameter on 3rd day post of aneurysm establish and 14rd day post of aneurysm establish respectively in the eSHE0.5 and eSHE1 group (n = 5 in each group). ns= no significance. Data were presented as mean ± s.d. Two-tailed paired t test (D) was used for comparing the diameter difference on the 3rd day and 14th day post of aneurysm establishment in SHE0.5 and SHE1 groups. ns = no significance. Source data are provided as a Source Data file. eSHE0.5, aneurysm established in SHE 0.5 group; eSHE1, aneurysm established in SHE 1 group.

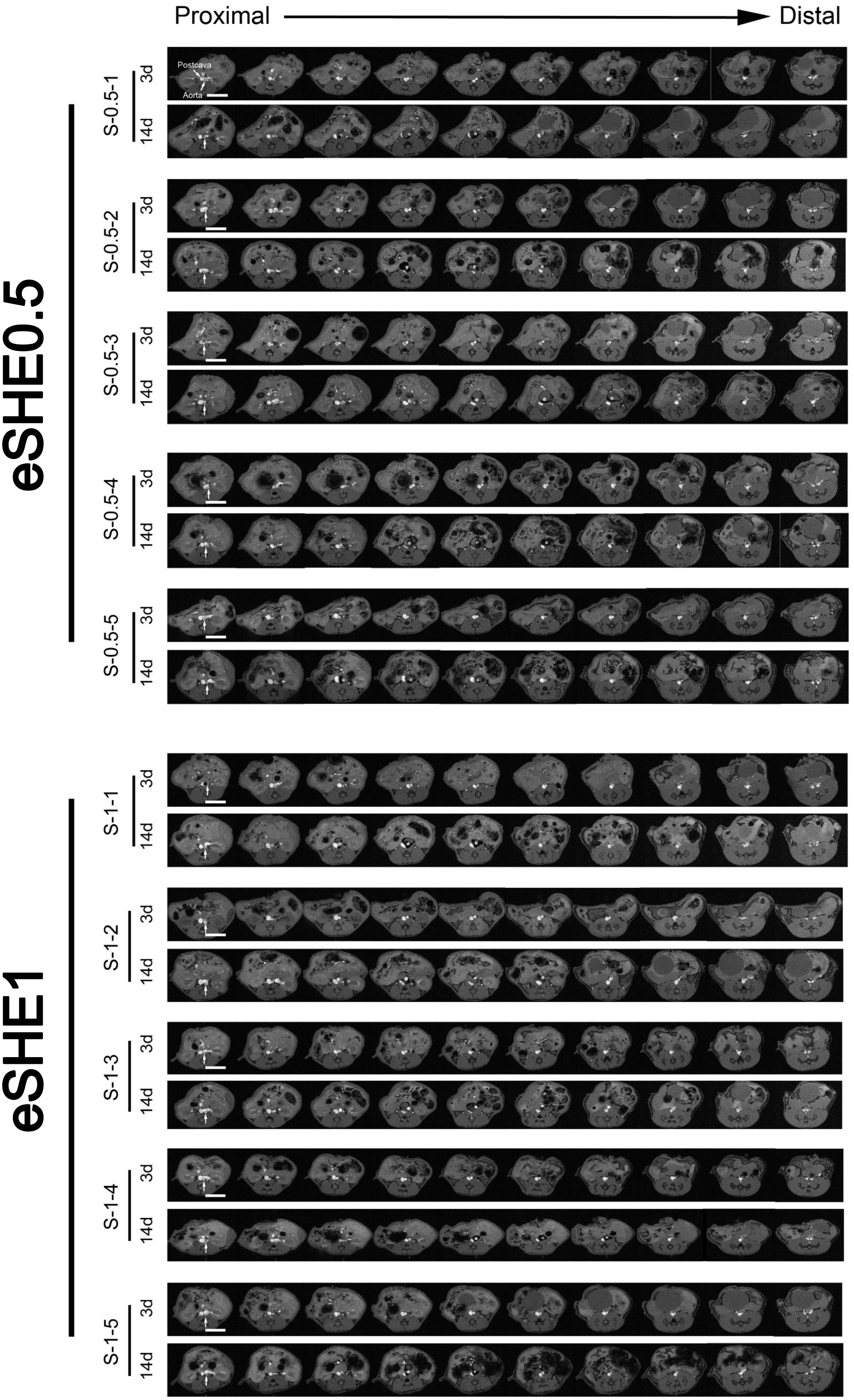

Supplementary Figure 16. MRI imaging summary of aorta in the eSHE0.5 and eSHE 1 group.

The vessel marked with white arrow is aorta. Scale bar = 5mm.

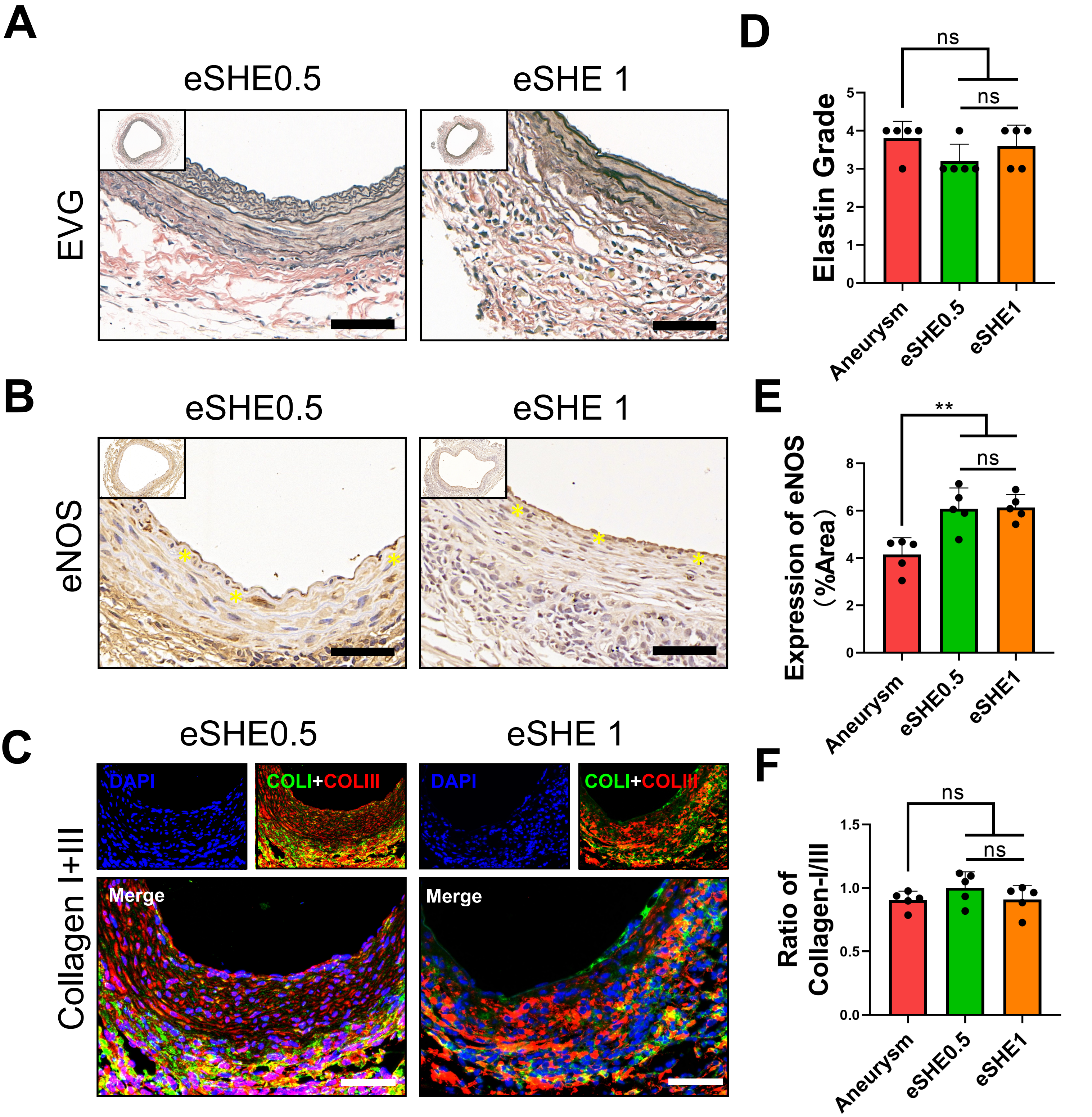

Supplementary Figure 17. EVG, eNOS and collagen staining of aorta in established aneurysm wrapping groups.

(A) Transverse sections of aorta were stained EVG in eSHE1 and eSHE0.5 groups. (B) Transverse sections of aorta were stained eNOS in eSHE1 and eSHE0.5 groups. The positive areas were marked with yellow *. (C) Transverse sections of aorta were stained collagen I/III in eSHE1 and eSHE0.5 groups. (D through F) The statistical histogram of elastin grade, eNOS expression and ratio of collagen-I/III respectively in aneurysm, eSHE0.5 and eSHE1 group (n = 5 in each group). Scale bar = 60 μm. Data were presented as mean ± s.d. Kruskal-Wallis test with Dunn's multiple comparisons test (D) was used for elastin grading. Elastin grade (D): Aneurysm group compared to eSHE0.5 group, ns $p = 0.1986$; Aneurysm group compared to eSHE1group, ns $p > 0.9999$; eSHE0.5group compared to eSHE1 group, ns $p = 0.662$. Ordinary one-way ANOVA test with Tukey's multiple comparisons test (E, F) was used for evaluation of eNOS expression and collagen ratio in groups. eNOS expression (E): eSHE0.5 and eSHE1 group compared to Aneurysm group, **$p = 0.0031$ and 0.0026 respectively. eSHE0.5 group compared to eSHE1 group, ns $p = 0.9942$. Collagen ratio (F): eSHE0.5 and eSHE1 group compared to Aneurysm group, ns $p =$ 0.3288 and 0.9939 respectively; eSHE0.5 compared to eSHE1, ns $p = 0.3782$. Source data are provided as a Source Data file. EVG, Verhoeff's van Gieson staining.

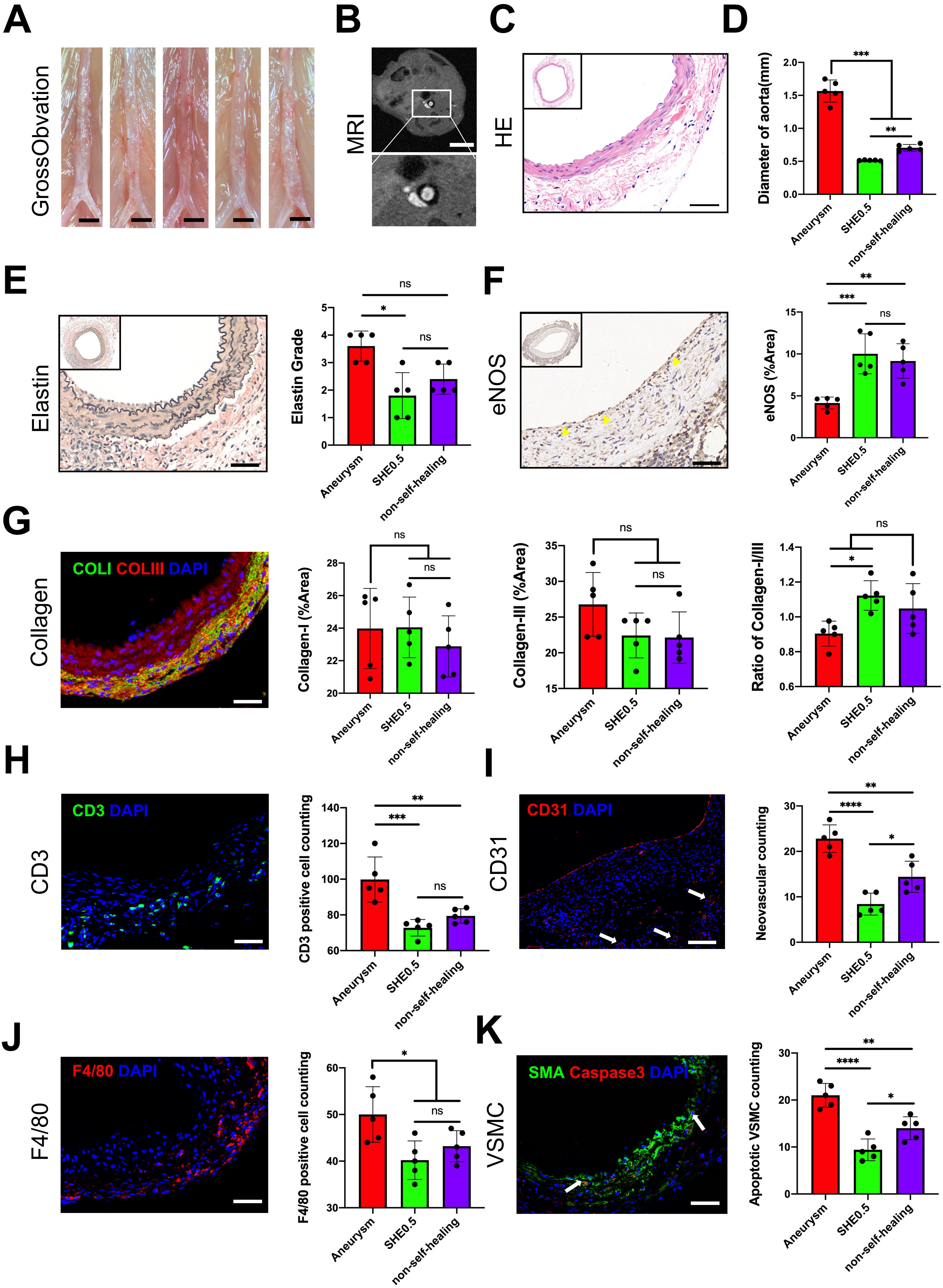

A
GrossObvation
B
MRI
C
HE
D
Diameter of aorta(mm)
Aneurysm
SHE0.5
non-self-healing
E
Elastin
Elastin Grade
F
eNOS
eNOS (%Area)
G
Collagen
COLI COLIII DAPI
Collagen-I (%Area)
Collagen-III (%Area)
Ratio of Collagen-I/III
H
CD3
CD3 DAPI
CD3 positive cell counting
I
CD31
CD31 DAPI
Neovascular counting
J
F4/80
F4/80 DAPI
F4/80 positive cell counting
K
VSMC
SMA Caspase3 DAPI
Apoptotic VSMC counting

Supplementary Figure 18. Comparison of limiting effect against aneurysm progress between SHE0.5 and non-self-healing material.

The silicone rubber was used as non-self-healing material to wrap aneurysm. (A) Grossobvation image of aorta after14 days wrapping by silicone rubber. Scale bar = 1mm (B) The representative MRI scanning image of aorta after14 days wrapping by silicone rubber. Scale bar = 5mm. (C) Transverse sections of aorta were stained with hematoxylin-eosin in non-self-healing material group, Scale bar = 50 µm. (D) The statistic histogram of aorta diameter on day 14 post of operation in aneurysm, SHE0.5 and non-self-healing material group. (E) Transverse sections of aorta were stained EVG in non-self-healing material groups and the statistic histogram of elastin grade in aneurysm, SHE0.5 and non-self-healing material group. (F)Transverse sections of aorta were stained eNOS in non-self-healing material group and the statistic histogram of eNOS positive area percentage in aneurysm, SHE0.5 and non-self-healing material group. The positive areas were marked with yellow triangle. (G) Transverse sections of aorta were stained collagen I/III in non-self-healing material group and the statistic histograms of several collagen content in aneurysm, SHE0.5 and non-self-healing material group. Scale bar = 40 µm. (H) Transverse sections of aorta were stained CD3 in non-self-healing material group and the statistic histograms of CD3 positive cell counting per high power field in aneurysm, SHE0.5 and non-self-healing material group. Scale bar = 40 µm. (I) Transverse sections of aorta were stained CD31 in non-self-healing material group and the statistic histograms of neo-vessel counting per high power field in aneurysm, SHE0.5 and non-self-healing material group. Scale bar = 40 µm. (J) Transverse sections of aorta were stained F4/80 in non-self-healing material group and the statistic histograms of F4/80 positive cell counting per high power field in aneurysm, SHE0.5 and non-self-healing material group. Scale bar = 40 µm. (K) Transverse sections of aorta were dual labeled with α-SMA and caspase-3 in non-self-healing material group and the statistic histograms of apoptotic vascular smooth muscle cell counting per high power field in aneurysm, SHE0.5 and non-self-healing material group. Scale bar = 40 µm. (n = 5 in each group). Data were presented as mean ± s.d. Brown-Forsythe ANOVA test with Dunnett's multiple comparison test (D) was used for comparing diameter of aorta in groups. Diameter of aorta (D): SHE0.5 and non-self-healing group compared to Aneurysm group, ***p = 0.0004 and 0.0003 respectively; SHE0.5 group compared to non-self-healing group, **p = 0.0028. Kruskal-Wallis test with Dunn's multiple comparisons test (E) was used for elastin grading. Elastin grade (E): Aneurysm group compared to SHE0.5 group, *p = 0.0121; Aneurysm and SHE0.5 group compared to non-self-healing group, ns p = 0.1395 and p > 0.9999 repectively. Ordinary one-way ANOVA test with Tukey's multiple comparisons test (F, G, H, J, K,) was used for evaluation of eNOS expression, collagen expression, CD3 cell counting, F4/80 positive cell counting, apoptotic VSMC counting and neovascular counting in groups. eNOS expression (F): Aneurysm group compared to SHE0.5 gruop, ***p = 0.0009; Aneurysm group compared to non-self-healing group, **p = 0.003; SHE0.5 group compared to non-self-healing group, ns p = 0.7532. Ratio of collagen I/III (G): Aneurysm group compared to SHE0.5 group, *p = 0.0159; Aneurysm and SHE0.5 group compared to non-self-healing, ns p = 0.1146 and 0.5181 respectively. CD3 positive cell counting (H): Aneurysm group compared to SHE0.5 group, ***p = 0.0005; Aneurysm group compared to non-self-healing group, **p = 0.0048; SHE0.5 group compared to non-self-healing group, ns p = 0.4285. CD31 positive neovessel counting (H): Aneurysm group compared to SHE0.5 group, ****p < 0.0001; Aneurysm group compared to non-self-healing group, ** p = 0.0021; SHE0.5 group compared to non-self-healing group, * p = 0.0203; F4/80 positive cell counting (J): Aneurysm group compared to SHE0.5 group, *p = 0.0146; Aneurysm and SHE0.5 group compared to non-self-healing group, ns p = 0.0897 and 0.5743 respectively. Apoptotic VSMC counting: Aneurysm group compared to SHE0.5 group, ****p < 0.0001; Aneurysm group compared to non-self-healing group, **p = 0.0018; SHE0.5 group compared to non-self-healing, *p = 0.0284. Source data are provided as a Source Data file. VSMC, vascular smooth muscle cell.

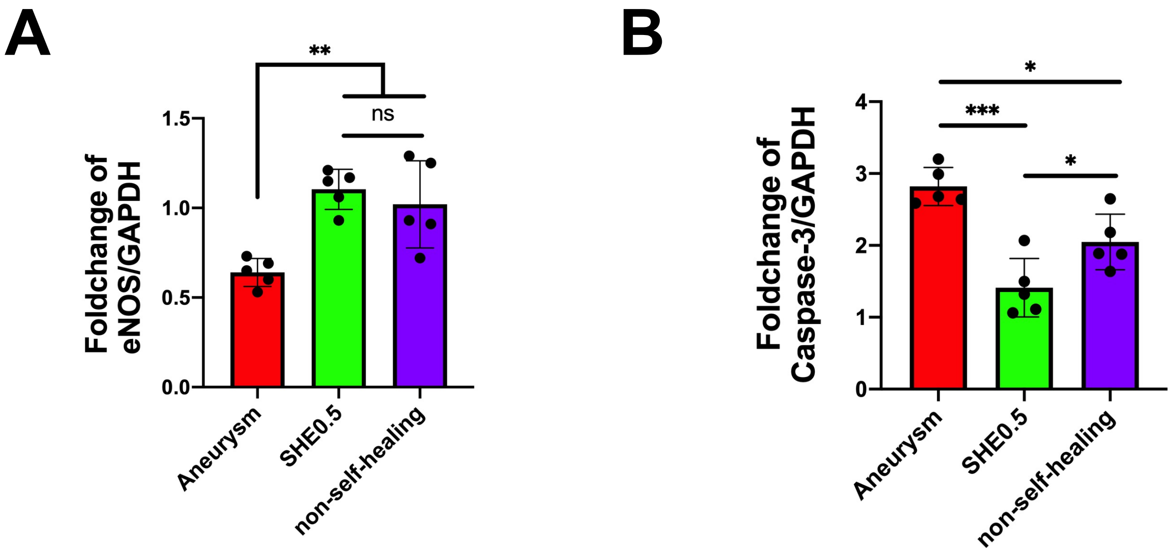

Supplementary Figure 19. Comparison of mRNA Transcriptional changes between SHE0.5 and non-self-healing material after aneurysm wrapping.

(A) The statistical histogram of eNOS mRNA transcriptional changes in sham, aneurysm, SHE1 and SHE0.5 group (n = 5 in each group). GAPDH was used as internal reference. ** P < 0.01, ns= no significance. (B) The statistical histogram of caspase-3 mRNA transcriptional changes in sham, aneurysm, SHE1 and SHE0.5 group (n = 5 in each group). GAPDH was used as internal reference. Data were presented as mean ± s.d. Ordinary one-way ANOVA test with Tukey's multiple comparisons test (A, B) was used for comparing the difference of eNOS transcription foldchange and caspase-3 transcription foldchange in groups. Foldchange of eNOS transcription (A):SHE0.5 and non-self-healing group compared to Aneurysm group, **p = 0.0017 and 0.0074 respectively; SHE0.5 group compared to non-self-healing group, ns p = 0.6947. Foldchange of caspase-3 transcription (B): Aneurysm group compared to SHE0.5 group, ***p = 0.0001; Aneurysm and SHE0.5 group compared to non-self-healing group, *p = 0.0392. Source data are provided as a Source Data file. GADPH, glyceraldehyde 3-phosphate dehydrogenase.

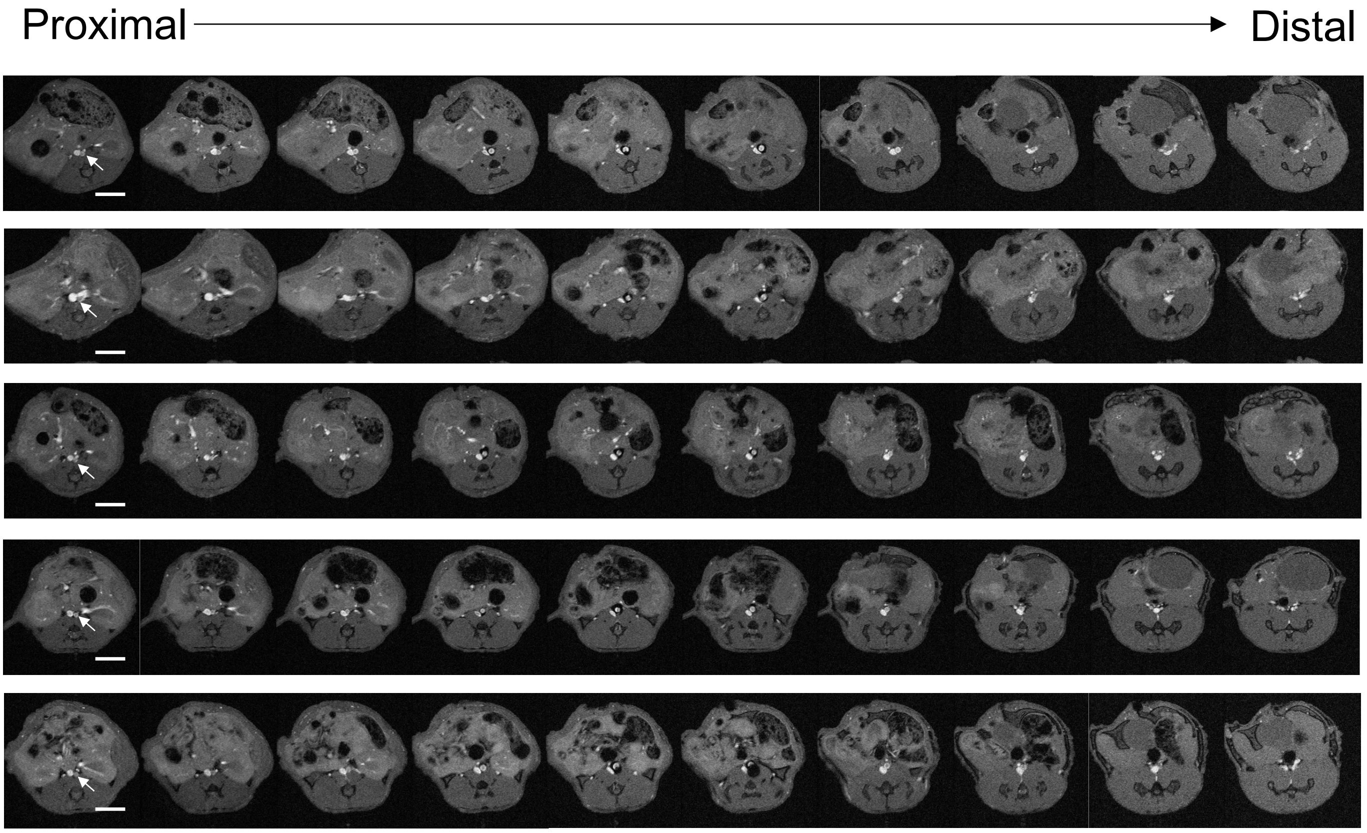

Supplementary Figure 20. MRI imaging summary of aorta in non-self-healing material group.

The vessel marked with white arrow is abdominal aorta.

Scale bar = 5mm.

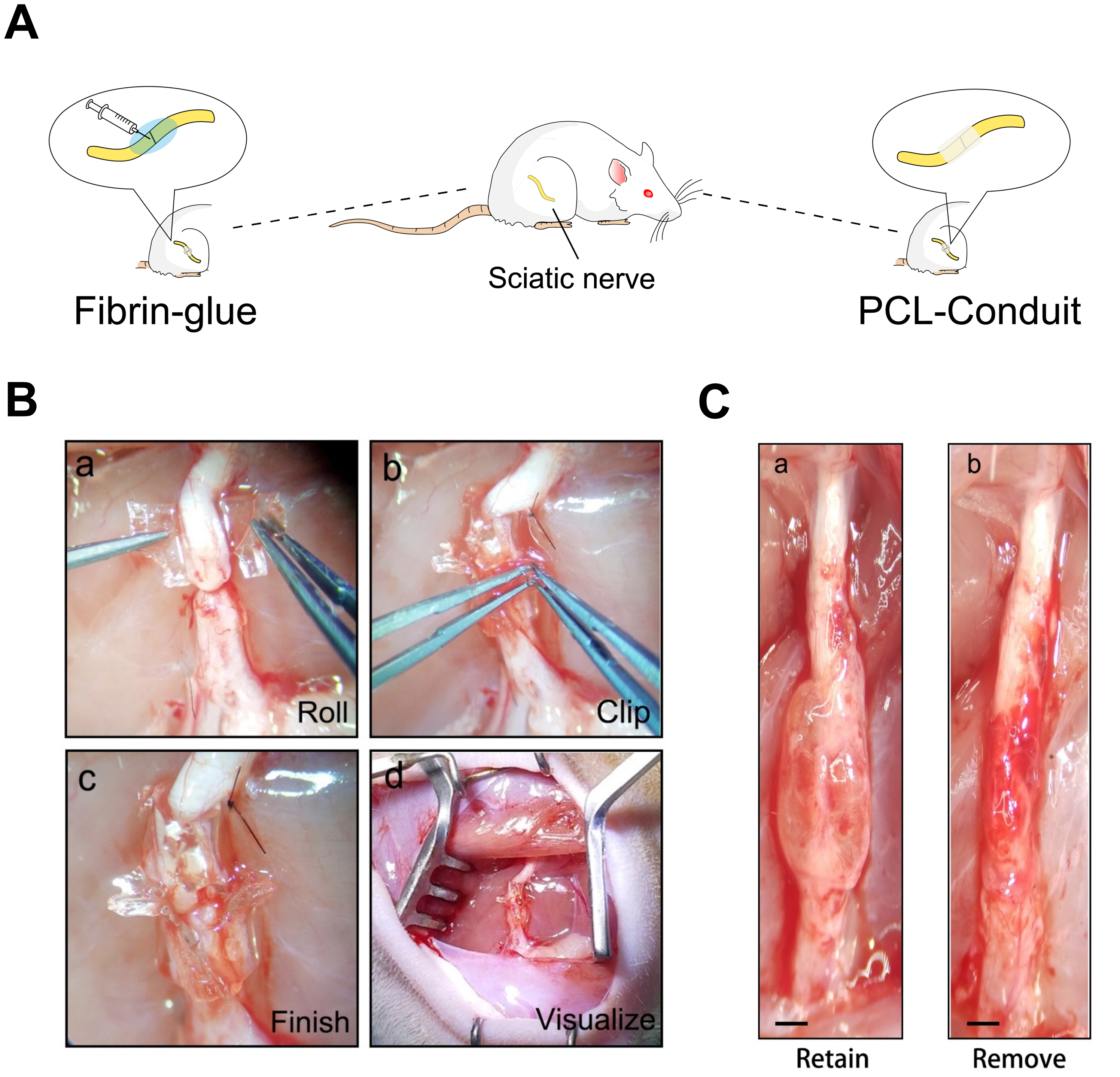

Supplementary Figure 21. Pattern of study design, operation procedure of SHE anastomosis and Gross observation on 6 weeks post of SHE anastomosis.

(A) Pattern diagram of study design. (B-a and B-b) The pre-shaped LEGO like SHEs were wrapped and healed around the two ends of nerve respectively, and the two "foot" of each SHE were healed together by clipping bringing the junction of nerve. (B-c and B-d) Partial and overall close-up view after anastomosis. (C-a) The gross observation shows the residual SHE around sciatic nerve. The surface of SHE has been polished smoothly by friction between muscles. (C-b) The gross observation shows the anastomotic sciatic nerve after removing the SHE. The signs of bleeding around nerve also indirectly indicate the presence of unobstructed vessels on the surface of nerve. Scale bar = 1mm.

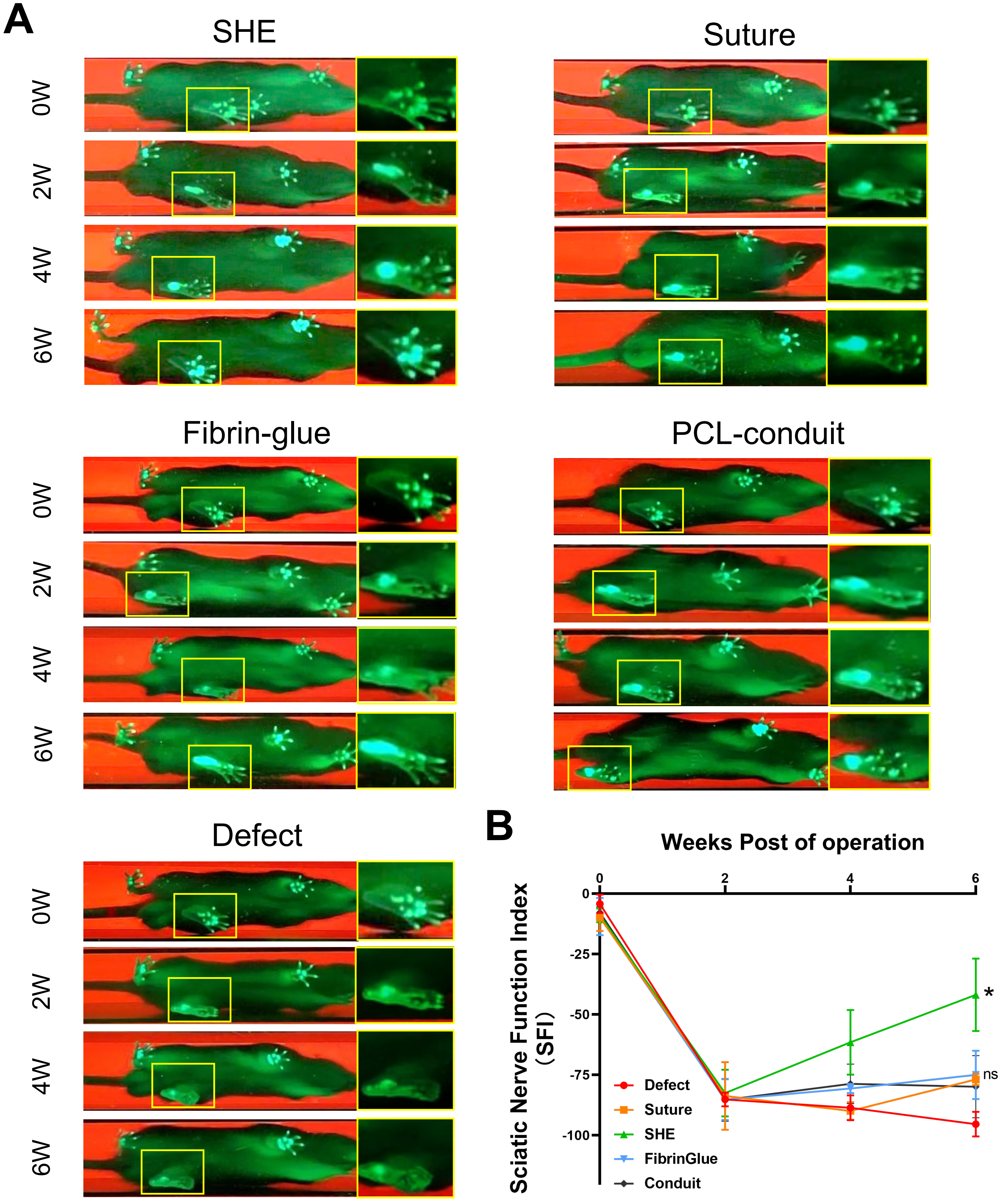

Supplementary Figure 22. Gait analysis and statistical histogram of SFI .
(A) The gait analysis in SHE, suture, fibrin-glue, PCL-conduit and defect group at the time of before surgery 0w , 2w, 4w, 6w post of surgery respectively. The foot prints of experiment side were highlighted by the yellow solid line. (B) Statistical histogram of sciatic nerve function index (n = 5 in each group). Data were presented as mean ± s.d. Ordinary one-way ANOVA test with Tukey's multiple comparisons test (B) was used for evaluation of Sciatic nerve function index. After 6 weeks, Suture, FibrinGlue and Conduit group compared to SHE group, *p = 0.0355. No significant difference among suture, fibringlue and conduit group. Source data are provided as a Source Data file. W, week.

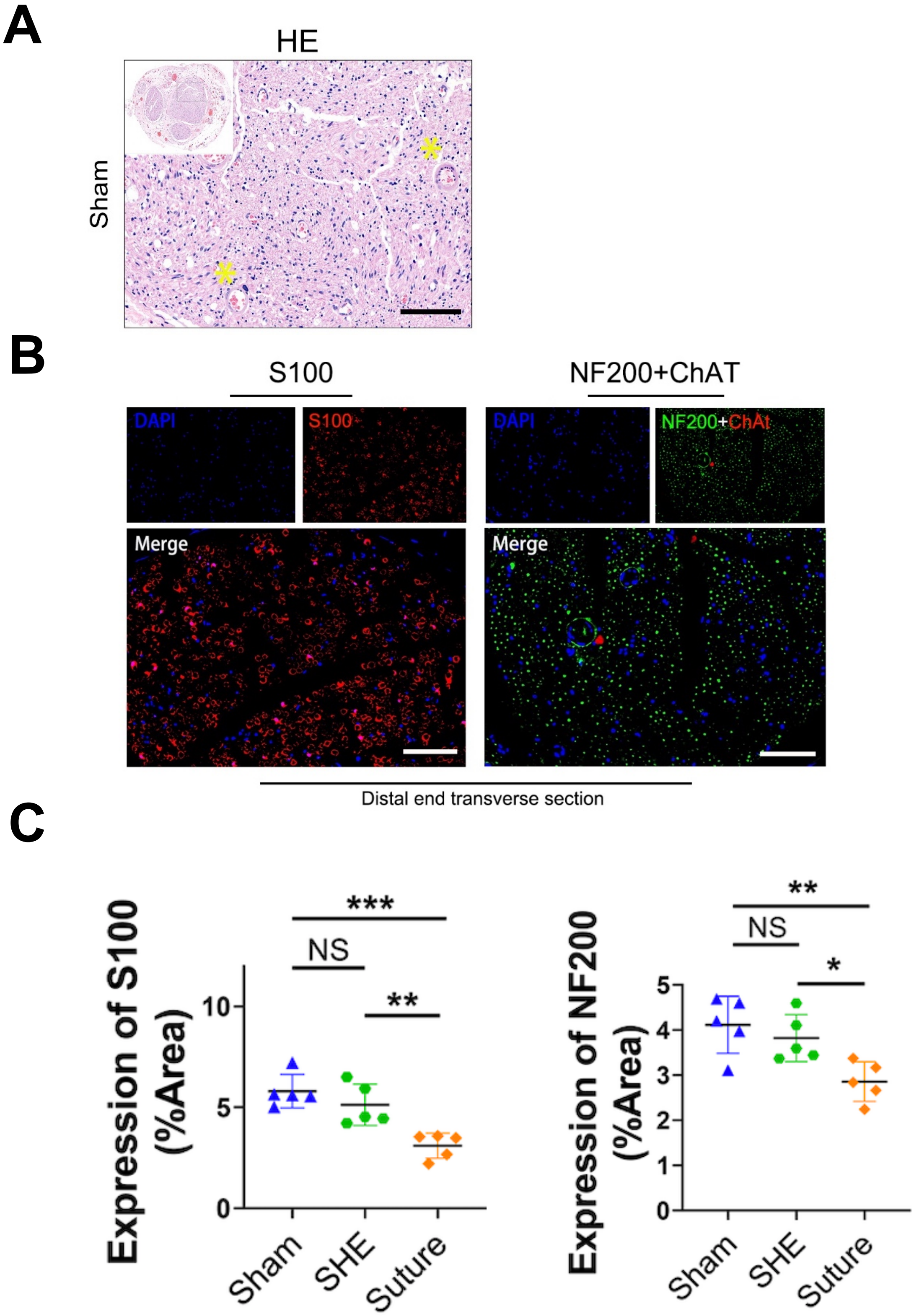

Supplementary Figure 23. HE and immunofluorescence staining of sciatic nerve in sham group.

(A) HE staining of transverse section of sciatic nerve in sham group. The structures marked with yellow * are capillaries. (B) S100 and NF200+ChAT immunofluorescence staining of distal end transverse section of sciatic nerve in sham group. (C) Statistical plot of S100 and NF200 expression respectively per section in sham, SHE and suture group (n = 5 in each group).Static plot: *P < 0.05, ** P < 0.01, *** P < 0.001, ****P < 0.00, ns = no significance. Scale bar=100μm. Data were presented as mean ± s.d. Ordinary one-way ANOVA test with Tukey's multiple comparisons test (C) was used for analysis of S100 expression and NF200 expression. Expression of S100: Sham compared to SHE, ns p = 0.4356; Sham compared to Suture, ***p = 0.0007; SHE compared to Suture, **p = 0.0065; Expression of NF200: Sham compared to SHE, ns p = 0.6702; Sham compared to Suture, ** p = 0.0078; SHE compared to Suture, * p = 0.0369. Source data are provided as a Source Data file.

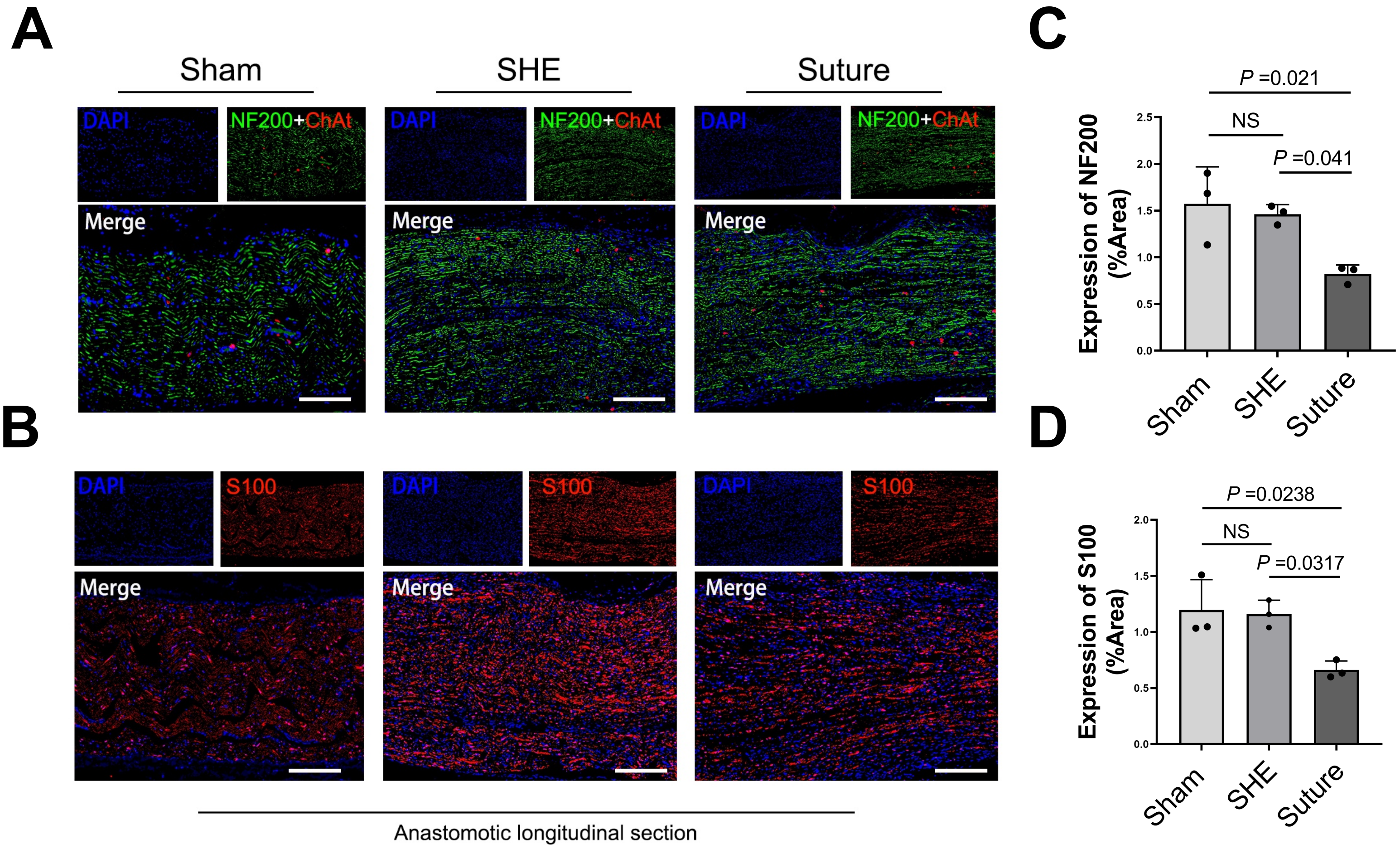

Supplementary Figure 24. NF200+ChAT and S100 immunofluorescence staining of anastomotic longitudinal section of sciatic nerve.

(A) NF200+ChAT immunofluorescence staining of anastomotic longitudinal section of sciatic nerve in sham, SHE, suture group. The positive axon markers (green) were arranged linearly along the nerve axis, which directly proved that the nerve axis at the anastomosis was continuous. (C) Statistical histogram of NF200 per section in sham, SHE and suture group (n = 3 in each group). Scale bar = 200μm. (B) S100 immunofluorescence staining of anastomotic longitudinal section of sciatic nerve in sham, SHE, suture group. The positive markers (red) were arranged linearly along the nerve axis, which indirectly proved that the nerve axis at the anastomosis was continuous. (D) Statistical histogram of S100 per section in sham, SHE and suture groups (n = 3 in each group). Scale bar = 200 μm. Data were presented as mean ± s.d. Ordinary one-way ANOVA test with Tukey's multiple comparisons test (C, D) was used for analysis of S100 expression and NF200 expression in anastomotic longitudinal section of each group. NS = no significance. Source data are provided as a Source Data file.

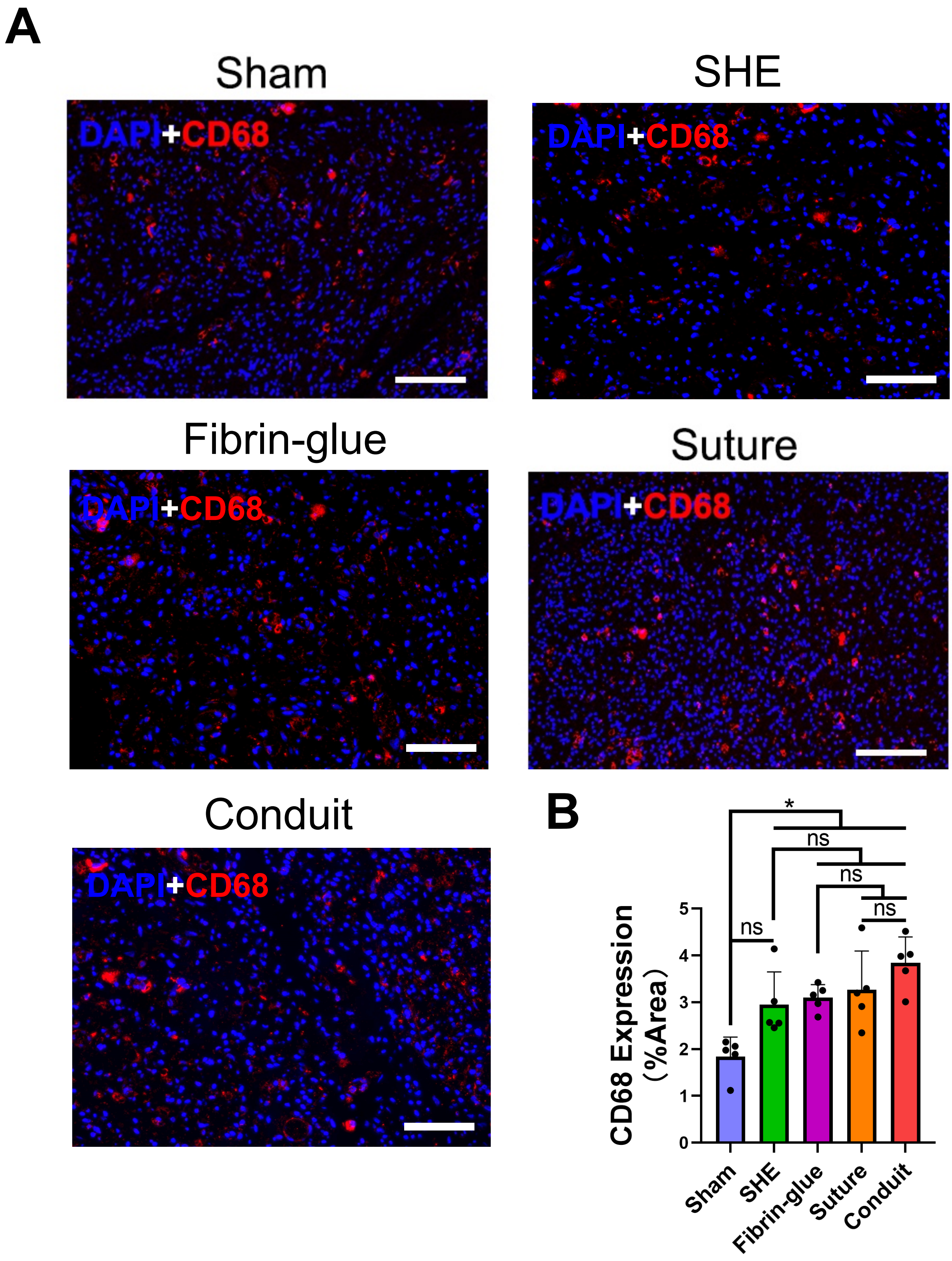

Supplementary Figure 25. CD68 immunofluorescence staining for evaluation of inflammation response.

(A) CD68 immunofluorescence staining of sciatic nerve in sham, SHE, Fibrin-glue, suture and conduit group at week 6 post of surgery. The positive macrophage markers (Red) were presented on the cross sections. (B)The statistic histogram of CD68 expression among groups. (n = 5 in each group). Scale bar = 100μm. Data were presented as mean ± s.d. Ordinary one-way ANOVA test with Tukey's multiple comparisons test (B) was used for analysis of CD68 expression. SHE, Fibrin-glue, Suture and Conduit group compared to Sham group, *p = 0.022. Source data are provided as a Source Data file. ns = no significance.

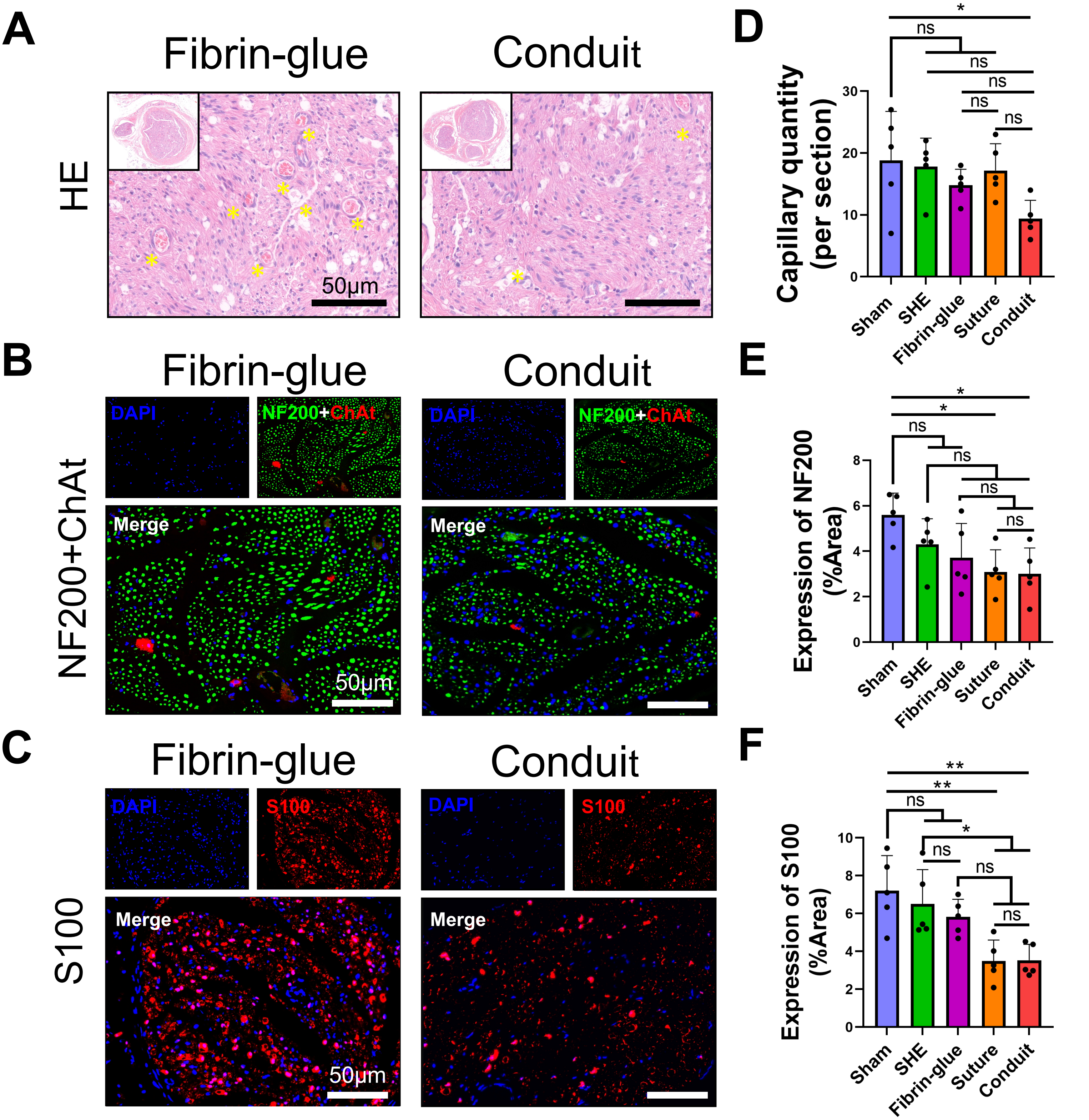

Supplementary Figure 26. HE and immunofluorescence staining of sciatic nerve in fibrin-glue and PCL-conduit group.

(A) HE staining of transverse section of sciatic. The structures marked with yellow * are capillaries. (B) NF200+ChAT immunofluorescence staining of distal end transverse section of sciatic nerve. (C) S100β immunofluorescence staining of distal end transverse section of sciatic nerve. (D through F) Statistical histogram of capillary number, NF200 expression and S100 expression respectively per section (n = 5 in each group). Scale bar = 50μm. Data were presented as mean ± s.d. Ordinary one-way ANOVA test with Tukey's multiple comparisons test (D, E, F) was used for analysis of capillary quantity, S100 expression, NF200 expression. Capillary quantity (D): Sham group compared to Conduit group, *p = 0.0443. No significant difference among SHE, fibrin-glue, suture and conduit group. Expression of NF200 (E): Suture and Conduit group compared to Sham group, *p = 0.02 and 0.0154 respectively. No significant difference between sham and SHE group and among SHE, fibrin-glue, suture and conduit group. Source data are provided as a Source Data file. ns = no significance.

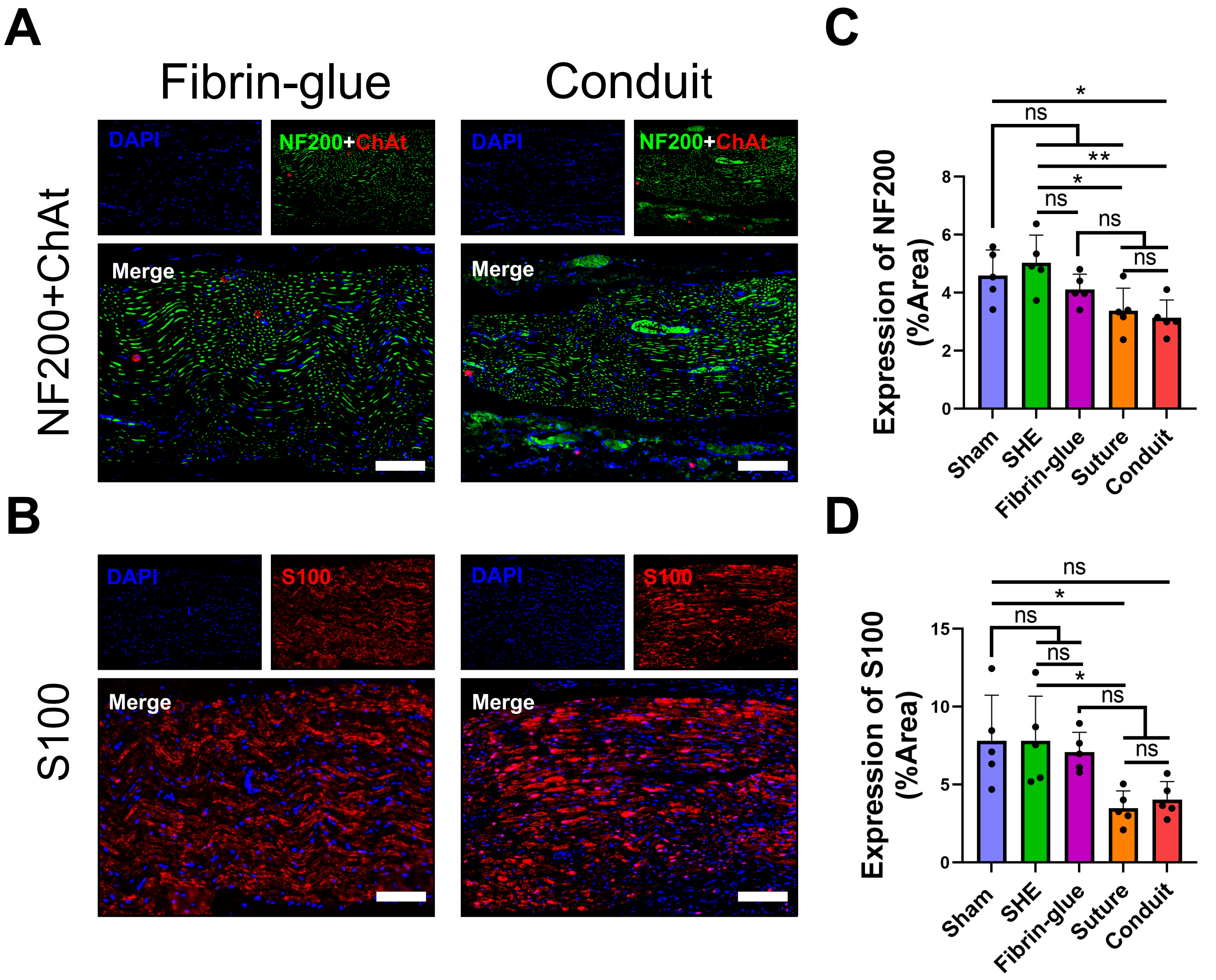

Supplementary Figure 27. Immunofluorescence staining of anastomotic longitudinal section of sciatic nerve in fibrin-glue and PCL-conduit group. (A) NF200+ChAT immunofluorescence staining of anastomotic longitudinal section of sciatic nerve. (B) S100β immunofluorescence staining of anastomotic longitudinal section of sciatic nerve. (C through D) Statistical histogram of NF200and S100 expression respectively per section (n = 5 in each group). Scale bar = 100μm. Data were presented as mean ± s.d. Ordinary one-way ANOVA test with Tukey's multiple comparisons test (C, D) was used for analysis of NF200 expression, S100 expression in anastomotic longitudinal section of each group. Expression of NF200 (C): Sham and SHE group compared to Conduit group, *p = 0.049 and 0.0208 respectively; SHE group compared to Conduit group, **p = 0.0071; SHE, fibrin-glue and Suture group compared to Sham group, ns p = 0.8959, 0.858 and 0.1267 respectively. SHE group compared to fibrin-glue, ns p = 0.3588. Suture and Conduit group compared to Fibrin-glue group, ns p = 0.5569 and 0.2948 respectively. Suture group compared to Conduit group, ns p = 0.9877. Expression of S100 (D): Sham and SHE group compared to Suture group, *p = 0.0238 and 0.0234 respectively. Sham, SHE, Fibrin-glue and Suture group, ns p = 0.0587, 0.0578, 0.1681 and 0.9921 respectively. SHE group compared to Fibrin-glue group, ns p = 0.979; Fibrin-glue group compared to Suture group, ns p = 0.075. Source data are provided as a Source Data file. ns = no significance.

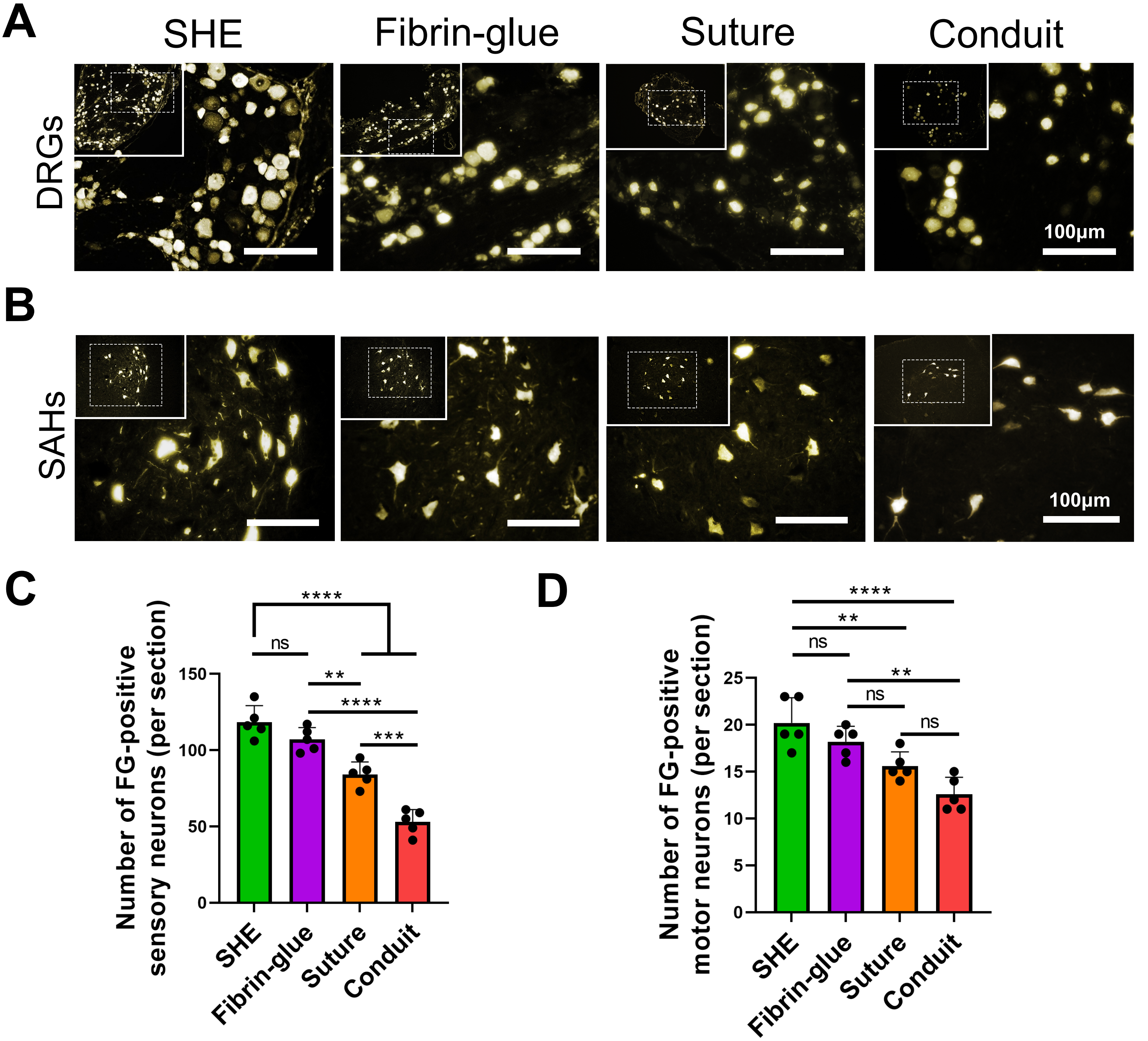

Supplementary Figure 28. Fluoro-gold (FG) retrograde tracing in SHE, fibrin-glue, suture and PCL-conduit group.

(A) Images of FG-labeled motor neurons in the sensory neurons in dorsal root ganglia (DRGs) at 6 weeks after surgery. (B) Images of FG-labeled motor neurons in the spinal anterior horns (SAHs) at 6 weeks after surgery. (C and D) The average number of FG-positive motor neurons and sensory neurons ($n = 5$ in each group). Scale bar = 100μm. Data were presented as mean ± s.d. Ordinary one-way ANOVA test with Tukey's multiple comparisons test (C, D) was used for analysis of FG-positive neurons counting in each group. Number of FG-positive sensory neurons counting (C): SHE group compared to Fibrin-glue group, ns $p = 0.2089$; Suture and Conduit group compared to SHE group, ****$p < 0.0001$; Fibrin-glue group compared to Suture group, *$p = 0.0041$; Fibrin-glue group compared to Conduit group, ****$p < 0.0001$; Suture group compared to Conduit group, ***$p < 0.0002$. Number of FG-positive motor neurons counting (D): SHE group compared to Fibrin-glue group, ns $p = 0.4209$; SHE group compared to Suture group, **$p = 0.0095$; SHE group compared to Conduit group, ****$p < 0.0001$; Fibrin-glue and Conduit group compared to Suture group, ns $p = 0.1988$ and 0.1152 respectively; Fibrin-glue compared to Conduit group, **$p = 0.019$. Source data are provided as a Source Data file. ns = no significance; FG, fluoro-gold; DRGs, dorsal root ganglions; SAHs, spinal cord anterior horns.

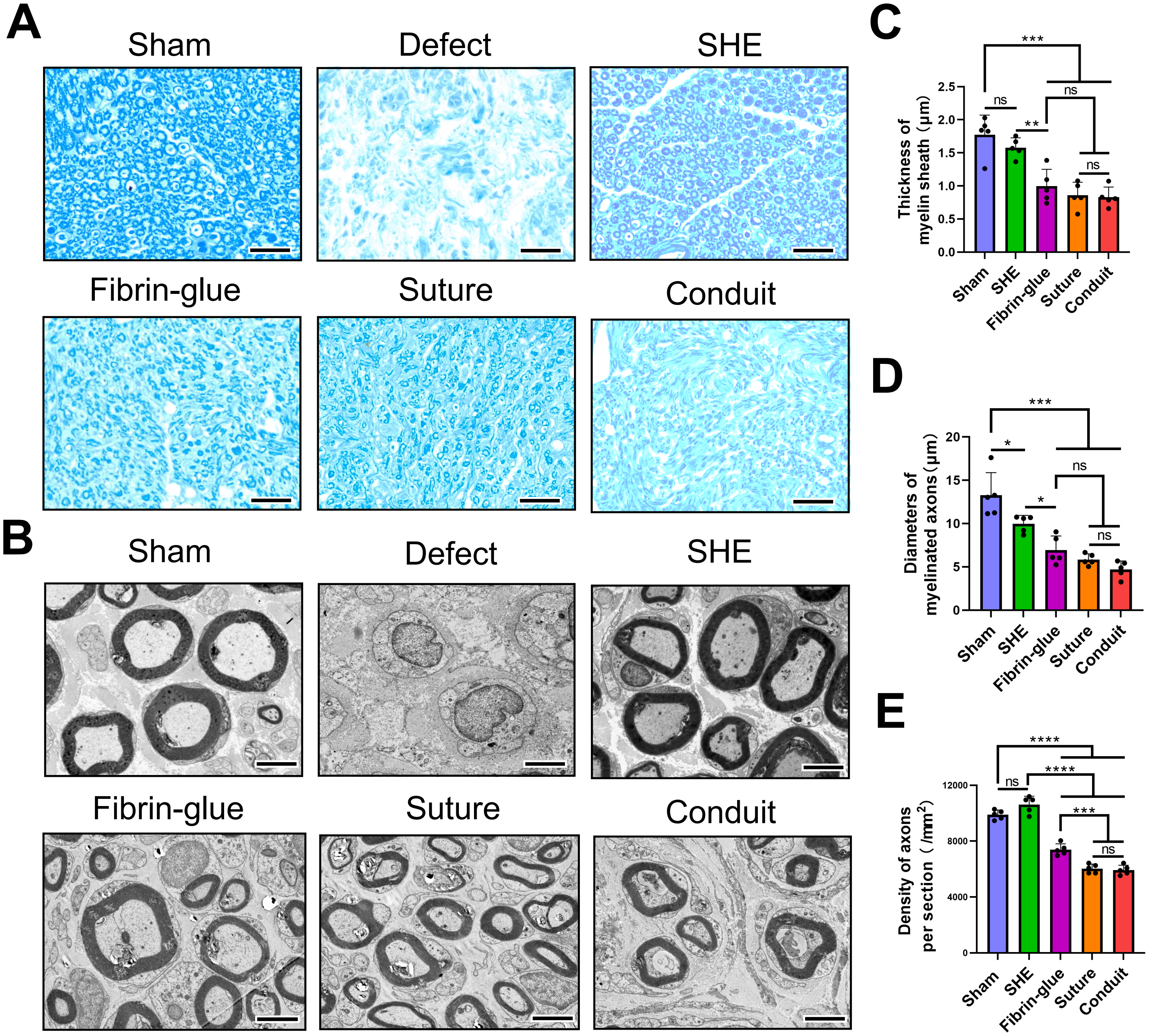

Supplementary Figure 29. LFB staining and transmission electron microscope (TEM) of sciatic nerve in sham, defect, suture, SHE, fibrin-glue and PCL-conduit group.

(A) Distal end of sciatic nerve imaginations of LFB staining in the sham, defect, suture, SHE, fibrin-glue and conduit group. Scale bar = 40μm. (B) Distal end of sciatic nerve imaginations of TEM in the sham, defect, suture, SHE, fibrin-glue and conduit group. Scale bar = 5μm. (C through E) The statistic histogram of myelin thickness, myelinated axons diameter and density of axons in the sham, SHE, suture, fibrin-glue conduit and defect group (n = 5 in each group). Data were presented as mean ± s.d. Ordinary one-way ANOVA test with Tukey's multiple comparisons test (C, D, E) was used for analysis of myelin sheath thickness, myelinated axons diameter and axons density. Thickness of myelin sheath (C): Sham group compared to SHE group, ns p = 0.6247; Sham group compared to Fibrin-glue group, ***p = 0.0001; Suture and Conduit group compared to Sham group, ****p < 0.0001; SHE group compared to Fibrin-glue group, **p = 0.0033; Suture and Conduit group compared to SHE group, p = 0.0004 and 0.0002 respectively; Suture and Conduit group compared to Fibrin-glue group, ns p = 0.861 and 0.7579 respectively; Suture group compared to Conduit group, ns p = 0.9995. Diameter of myelinated axons (D): Sham group compared to SHE group, *p = 0.0226; Fibrin-glue, Suture and conduit group compared to Sham group, ****p < 0.0001; SHE group compared to Fibrin-glue group, *p = 0.0364; SHE group compared to Suture group, **p = 0.0031; SHE group compared to Conduit group, ***p = 0.0002; Suture and Conduit group compared to Fibrin-glue group, ns p = 0.7915 and 0.1908 respectively; Suture group compared to Conduit group, ns p = 0.7734. Diameter axons (E): Sham group compared to SHE group, ns p = 0.0738; Fibrin-glue, Suture and Conduit group compared to Sham group, ****p < 0.0001; Fibrin-glue, Suture and Conduit group compared to SHE group, ****p < 0.0001; Suture and Conduit group compared to Fibrin-glue group, ***p = 0.0004 and 0.0002 respectively; Suture group compared to Conduit group, ns p = 0.9936. Source data are provided as a Source Data file. ns = no significance.

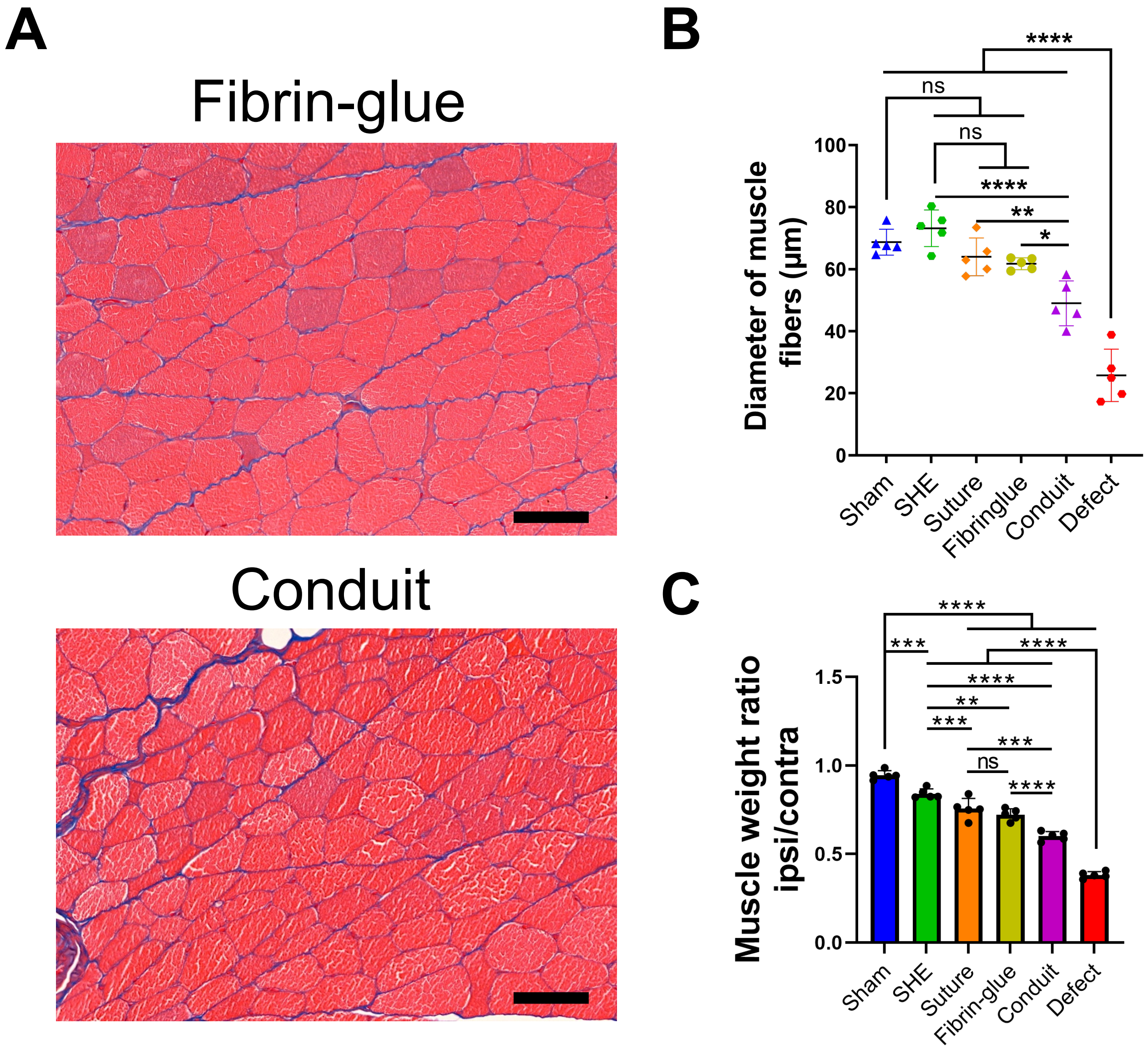

Supplementary Figure 30. Masson staining for evaluation muscle fibers.

(A) Masson staining of gastrocnemius muscle in fibrin-glue and PCL-conduit group at week 6 post of surgery. Scale bar = 50μm. (B)The statistic histogram of muscle fibers diameter in the sham, SHE, suture, fibrin-glue, conduit and defect group. (n = 5 in each group, 3fields of view per slice). (C)The statistic histogram of muscle weight ratio ipsi/contra in the sham, SHE, suture, fibrin-glue, conduit and defect group. (n = 5 in each group). Data were presented as mean ± s.d. Ordinary one-way ANOVA test with Tukey's multiple comparisons test (B, C) was used for analysis of muscle fiber diameter and muscle weight ratio. Diameter of muscle fiber (B): SHE, Suture and Fibrin-glue group compared to Sham group, ns p = 0.844, 0.8095 and 0.4647 respectively. Sham group compared to Conduit group, ***p = 0.0003; Sham group compared to Defect group, ****p < 0.0001; Suture and Fibrin-glue group compared to SHE group, ns p = 0.1882 and 0.0598 respectively; Conduit and Defect group compared to SHE group, ****p < 0.0001; Suture group compared to Fibrin-glue group, ns p = 0.991; Suture group compared to Conduit group, **p = 0.0072; Suture group compared to Defect group, ****p < 0.0001; Fibrin-glue group compared to Conduit group, *p = 0.0282; Fibrin-glue and Conduit group compared to Defect group, ****p < 0.0001. Muscle weight ratio (C): Sham group compared to SHE group, ***p = 0.0008; Suture, Fibrin-glue, Conduit and Defect group compared to Sham group, ****p < 0.0001; SHE group compared to Suture group, **p = 0.0077; SHE group compared to Fibrin-glue group, ***p = 0.0002; Conduit and Defect group compared to SHE group, ****p < 0.0001; Suture group compared to Fibrin-glue group, ns p = 0.6188; Conduit and Defect group compared to Suture group, ****p < 0.0001; Fibrin-glue group compared to Conduit group, ***p = 0.0001; Fibrin-glue and Conduit group compared to Defect group, ****p < 0.0001. Source data are provided as a Source Data file. ns = no significance.

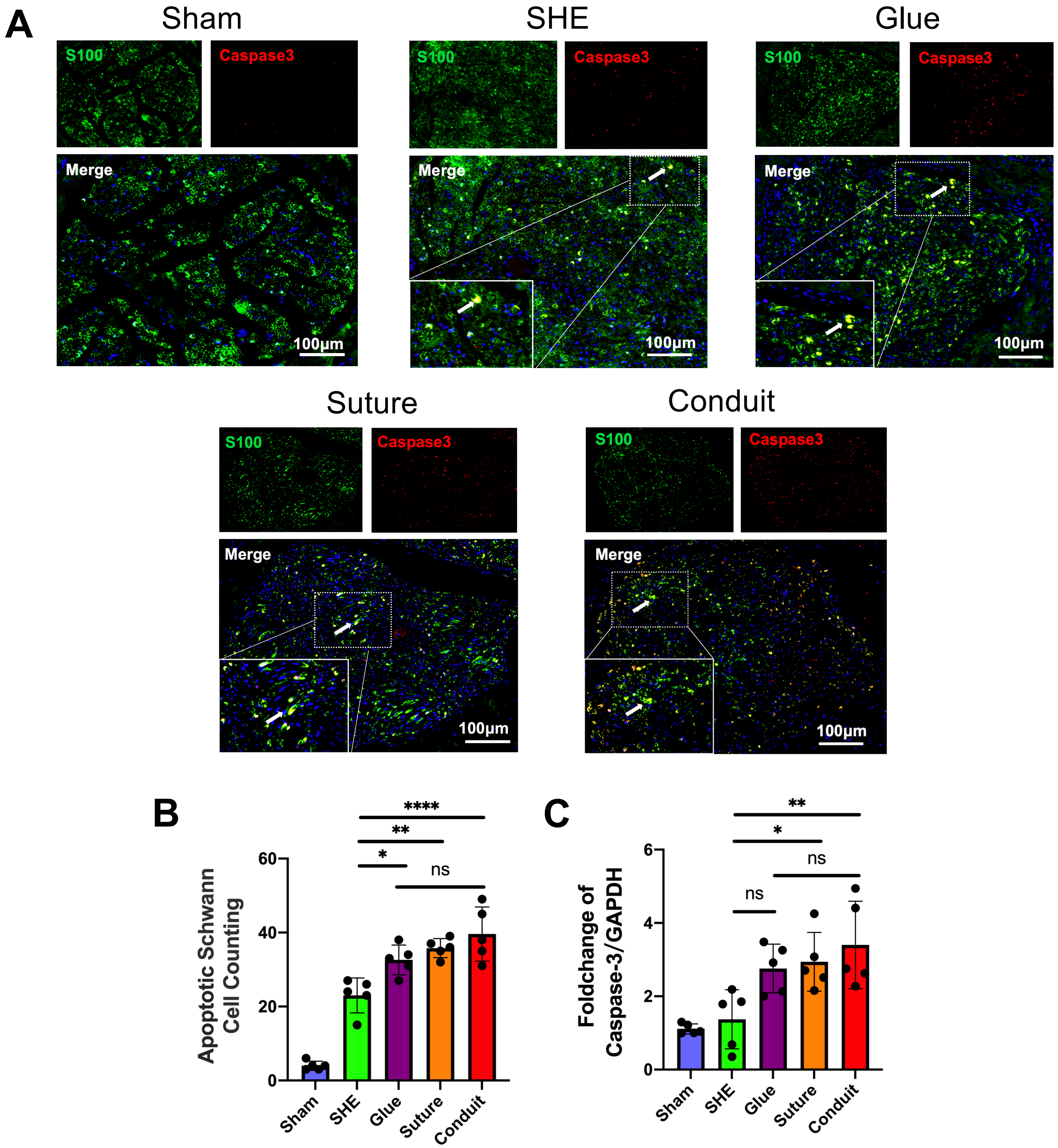

Supplementary Figure 31. Apoptosis of Schwann cell and mRNA transcription of caspase-3.

(A) Immunofluorescence staining of S100 and caspase-3 in SHE, glue, suture and conduit groups. Green represents S100 positive Schwann cell and red represents caspase-3 positive cell. White arrows illustrate the typical caspase-3 positive Schwann cells which are mixed with both green and red. Scale bar = 100 μm. (B) Statistic histogram of counting number of apoptotic Schwann cell based on images of immunofluorescence staining in sham, SHE, glue, suture and conduit groups. (C) Statistic histogram of mRNA relative expression foldchange of caspase-3 based on results of quantitative real-time PCR in sham, SHE, glue, suture and conduit groups. GAPDH was used as internal reference. (n = 5 in each group). Data were presented as mean ± s.d. Ordinary one-way ANOVA test with Tukey's multiple comparisons test (B, C) was used for analysis of apoptotic Schwann cell counting and caspase-3 transcription foldchange. Apoptotic Schwann cell counting (B): Glue group compared to SHE group, *p = 0.0221; Suture group compared to SHE group, **p = 0.0018; Conduit group compared to SHE group, ****p < 0.0001; No significant difference among Glue, Suture and Conduit group. Caspase-3 transcription foldchange (C): SHE group compared to Glue group, ns p = 0.0808; SHE group compared to Suture group, *p = 0.039; SHE group compared to Conduit group, **p = 0.0053; No significant difference among Glue, Suture and Conduit group. ns = no significance.

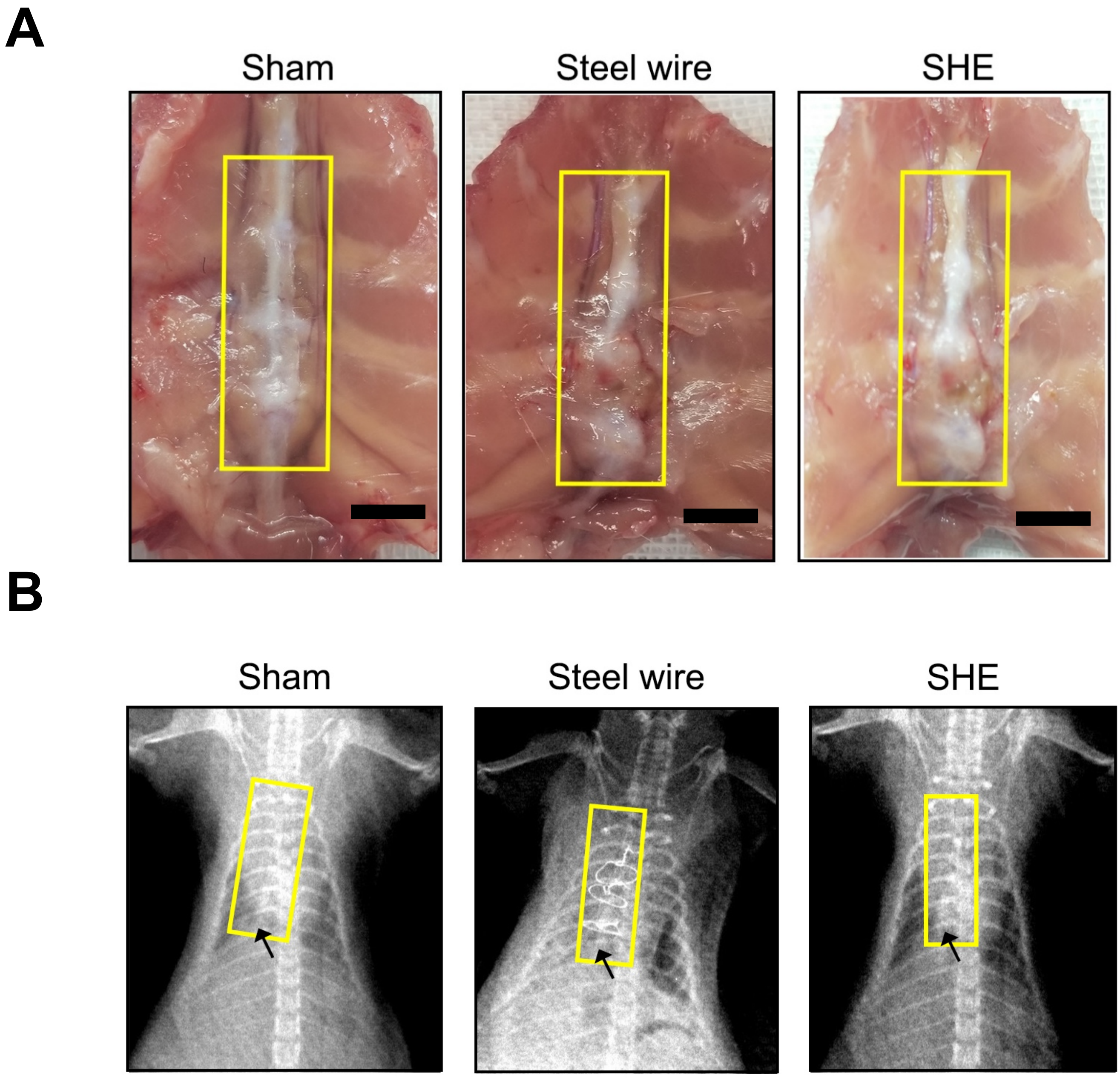

Supplementary Figure 32. Gross observation of sternum and Chest X-ray film in rat.

(A) The yellow solid line box shows the rat's sternum in sham, steel wire and SHE. The steel wire and SHE have been removed before photograph. Scale bar = 5mm. (B) Chest X-ray was performed on week 6 post of immobilization. The cord-like high-density shadow pointed by black arrow in the yellow solid line mine was the sternum of the rat. In the steel wire group, the high-density wire shadows could be seen.

Supplementary Tables

Supplementary Table1. Elastic Modulus Summary of materials used in this study

Material	Elastic Modulus	Application *in vivo*
SHE 0	172 ± 61 kPa	—
SHE 0.2	612 ± 199 kPa	Peripheral nerve coaptation
SHE 0.5	1166 ± 198 kPa	Aneurysm wrapping
SHE 1	1516 ± 227 kPa	
SHE 2	3724 ± 787 kPa	Sternum immobilization
Silicone rubber	2010 ± 291 kPa	As non-self-healing material control in aneurysm wrapping
PCL	154 ± 13 MPa	As non-self-healing nerve conduit control in peripheral nerve coaptation

Supplementary Table2. The names and sequences of primers used in this study

Study Part	Primer names	Primer sequences
**Aneurysm wrapping**	eNOS	S 5'-TGGACATCACTTCCCCGCCTA-3'
		A 5'- TGCCACTGAAGGAAATTGCTC-3'
	Caspase-3	S 5'-TGGAAAGCCGAAACTCTTCATCA-3'
		A 5'-CCACGACCCGTCCTTTGAAT-3'
	GAPDH	S 5'-CCTCGTCCCGTAGACAAAATG-3'
		A5'-TGAGGTCAATGAAGGGGTCGT-3'
**Peripheral nerve coaptation**	Caspase-3	S 5'- GGATTACCCTGAAATGGGCTTG-3'
		A 5'- ACAGGTCCGTTCGTTCCAAAA-3'
	GAPDH	S 5'- CTGGAGAAACCTGCCAAGTATG-3'
		A 5'- GGTGGAAGAATGGGAGTTGCT-3'
