# Supplementary material for: Self-healing polyurethane-elastomer with mechanical tunability for multiple biomedical applications in vivo
Source: Nat Commun. 2021 Jul 20;12:4395. doi: 10.1038/s41467-021-24680-x (PMC8292539; doi:10.1038/s41467-021-24680-x)
Supplement: Supplementary file 3 — Description of Additional Supplementary Files [file 41467_2021_24680_MOESM3_ESM.pdf]

Description of Additional Supplementary Files

File Name: Supplementary Movie 1
Description: **Self-healing property of the elastomer.** The self-healing elastomer can be easily rolled and wrapped. The edges of elastomer can be rapidly self-healed together at room temperature without chemical or physical stimulations.

File Name: Supplementary Movie 2
Description: **Operation of aorta wrapped with the elastomer:** After anesthesia and careful median laparotomy, the abdominal aorta was exposed from the renal vein to the iliac bifurcation before wrapped circumferentially by bibulous paper soaked with 10 µl of 100% elastase for 10 min and 0.9% saline flushing to stop reaction of elastase. In each SHEs groups, the SHEs were putted under abdominal artery before bended and wrapped the vessel until the edge of SHEs was healed together.

File Name: Supplementary Movie 3
Description: **Operation of sciatic nerve anastomosis by the elastomer:** after amputation, the proximal nerve and distal nerve were fixed on the muscle by suturing the adventitia of the nerve with one stitch respectively. The aim of fixing the nerve was to avoid axial shifting along the long axis after SHE0.2 intervention, and this process also reduced the tension between the stumps. After fixation, the pretrimmed SHEs were wrapped around the two ends of the nerve and allowed to heal, bridging the junction of the nerve with the presence of a small gap between the two ends of the nerve.

File Name: Supplementary Movie 4
Description: **Post of sternum immobilization by the elastomer on rat.** The SHE2s (2 mm × 2 mm × 20 mm), which were shaped into strips prior to the procedure, were traversed through intercostal muscles on both sides, and the two ends of the SHE were fixed and healed together to achieve sternum closure.

File Name: Supplementary Movie 5
Description: **(Spinal X-ray film) Before sternum immobilization by the elastomer on pig.** The yellow boxes indicated the unfixed sternum. The positions of spine and rib are marked by white arrows.

File Name: Supplementary Movie 6
Description: **(Spinal X-ray film) Post of sternum immobilization by elastomer on pig.** The yellow boxes indicated the fixed sternum. The positions of spine and rib are marked by white arrows.
