# Supplementary material for: Self-healing polyurethane-elastomer with mechanical tunability for multiple biomedical applications in vivo
Source: Nat Commun. 2021 Jul 20;12:4395. doi: 10.1038/s41467-021-24680-x (PMC8292539; doi:10.1038/s41467-021-24680-x)
Supplement: Supplementary file 10 — Reporting Summary [file 41467_2021_24680_MOESM10_ESM.pdf]

nature research

Corresponding author(s): Xiaofeng Ye

Last updated by author(s): Jun 3, 2021

Reporting Summary

Nature Research wishes to improve the reproducibility of the work that we publish. This form provides structure for consistency and transparency in reporting. For further information on Nature Research policies, see our Editorial Policies and the Editorial Policy Checklist.

Statistics

For all statistical analyses, confirm that the following items are present in the figure legend, table legend, main text, or Methods section.

n/a	Confirmed	
☐	☒	The exact sample size (*n*) for each experimental group/condition, given as a discrete number and unit of measurement
☐	☒	A statement on whether measurements were taken from distinct samples or whether the same sample was measured repeatedly
☐	☒	The statistical test(s) used AND whether they are one- or two-sided<br>*Only common tests should be described solely by name; describe more complex techniques in the Methods section.*
☒	☐	A description of all covariates tested
☐	☒	A description of any assumptions or corrections, such as tests of normality and adjustment for multiple comparisons
☐	☒	A full description of the statistical parameters including central tendency (e.g. means) or other basic estimates (e.g. regression coefficient) AND variation (e.g. standard deviation) or associated estimates of uncertainty (e.g. confidence intervals)
☐	☒	For null hypothesis testing, the test statistic (e.g. *F*, *t*, *r*) with confidence intervals, effect sizes, degrees of freedom and *P* value noted<br>*Give P values as exact values whenever suitable.*
☒	☐	For Bayesian analysis, information on the choice of priors and Markov chain Monte Carlo settings
☒	☐	For hierarchical and complex designs, identification of the appropriate level for tests and full reporting of outcomes
☒	☐	Estimates of effect sizes (e.g. Cohen's *d*, Pearson's *r*), indicating how they were calculated

Our web collection on statistics for biologists contains articles on many of the points above.

Software and code

Policy information about availability of computer code

Data collection	No software used
Data analysis	Sante MRI viewer 3.0<br>Image J version 1.51<br>Mircrosoft Excel version 2019<br>GraphPad Prism version 8<br>IBM SPSS statistic version 22

For manuscripts utilizing custom algorithms or software that are central to the research but not yet described in published literature, software must be made available to editors and reviewers. We strongly encourage code deposition in a community repository (e.g. GitHub). See the Nature Research guidelines for submitting code & software for further information.

Data

Policy information about availability of data

All manuscripts must include a data availability statement. This statement should provide the following information, where applicable:

- Accession codes, unique identifiers, or web links for publicly available datasets
- A list of figures that have associated raw data
- A description of any restrictions on data availability

All relevant data are available from the corresponding author upon reasonable request. All the data supporting the findings of this study are available within this article, supplementary information files.

April 2020

Field-specific reporting

Please select the one below that is the best fit for your research. If you are not sure, read the appropriate sections before making your selection.

☒ Life sciences ☐ Behavioural & social sciences ☐ Ecological, evolutionary & environmental sciences

For a reference copy of the document with all sections, see nature.com/documents/nr-reporting-summary-flat.pdf

Life sciences study design

All studies must disclose on these points even when the disclosure is negative.

Sample size	Based on the previous published research, we preformed sample size calculations on online platform-EDA (Experimental Design Assistant, national centre for the replacement refinement & reduction of animals in research, https://eda.nc3rs.org.uk/about). Following the instructions of EDA, we finished the experiment design diagram and typed the certain parameters which were based on results from published studies for power analysis. This was determined at a minimum of 5 mice for each group.
Data exclusions	No data were excluded from the analysis
Replication	Three technical replicates or more were performed for each experiments and experiments were repeated at least three times with similar results. All attempts at replication were successful.
Randomization	The animals were randomized between groups.
Blinding	Outcome assessors were blinded to group allocation.<br>Specifically:<br>First, allocation concealment: the investigators were unaware of the group to which the next animal taken from a cage will be allocated.<br>Second, blinded conduct of the experiment: animal caretakers were blinded to the allocation sequence. Even surgeons performed the certain operations, which they were aware of the specific allocation sequence, but they did not participate in outcomes assessing and communicate with investigators about allocation situations.<br>Last, blinded assessment of outcome: investigators assessing, measuring or quantifying experimental outcomes were blinded to the intervention.

Reporting for specific materials, systems and methods

We require information from authors about some types of materials, experimental systems and methods used in many studies. Here, indicate whether each material, system or method listed is relevant to your study. If you are not sure if a list item applies to your research, read the appropriate section before selecting a response.

Materials & experimental systems

n/a	Involved in the study	
☐	☒	Antibodies
☒	☐	Eukaryotic cell lines
☒	☐	Palaeontology and archaeology
☐	☒	Animals and other organisms
☒	☐	Human research participants
☒	☐	Clinical data
☒	☐	Dual use research of concern

Methods

n/a	Involved in the study	
☒	☐	ChIP-seq
☒	☐	Flow cytometry
☒	☐	MRI-based neuroimaging

Antibodies

Antibodies used

anti-Ly6G (1:100, ab25377, Abcam), rat monoclonal [clone name: RB6-8C5]
anti-F4/80 (1:100, ab90247, Abcam), rat monoclonal [clone name: F4/80]
anti-eNOS (1: 100, ab76198, Abcam), mouse monoclonal [clone name: M221]
anti-collagen I (1:100, ab6308, Abcam), mouse monoclonal [clone name: COL-1]
anti-collagen III (1:200, PA592066, Invitrogen), rabbit polyclonal
anti-NF200 (1:50, ab82259, Abcam), mouse monoclonal [clone name: N52]
anti-S100β (1:100, ab52642, Abcam), rabbit monoclonal [clone name: EP1576Y]
anti–ChAt (1:100, ab181023, Abcam), rabbit monoclonal [clone name: EPR13024(B)]
anti-caspase-3(1:100, 43-7800, Invitrogen), mouse monoclonal [clone name: 74T2]
anti-CD3(1:10, ab135372, Abcam), rabbit monoclonal [clone name: SP162]
anti-CD31(1:100, MA3100, Invitrogen), mouse monoclonal [clone name: HEC7]
Alexa Fluor 488 goat anti-mouse IgG (1:200, ab150117, Abcam)
Alexa Fluor 594 goat anti-rabbit IgG (1:200, ab150088, Abcam)
Alexa Fluor 594 goat anti-rabbit IgG (1:200, A32740, Invitrogen)

Validation	anti-Ly6G (1:100, ab25377, Abcam) - By manufacture immunohistochemistry (Frozen sections) analysis of Mouse frozen spleen tissue<br>anti-F4/80 (1:100, ab90247, Abcam)-By manufacture immunofluorescent staining of Mouse-derived Raw 264.7 cells<br>anti-eNOS (1:1000, ab76198, Abcam)-By manufacture western blotting analysis of Mouse placenta lysates and Human umbilical vein endothelial cells<br>anti-collagen I (1:100, ab6308, Abcam)- By manufacture immunoperoxidase staining of unfixed frozen tissue sections of human tonsil tissue<br>anti-caspase-3(43-7800, Invitrogen)- By manufacture immunohistochemistry analysis of Mouse colon tissue.<br>anti-CD3(1:10, ab135372, Abcam)- By manufacture flow cytometry analysis of Mouse splenocyte cell.<br>anti-CD31(1:100, MA3100, Invitrogen) - By manufacture immunofluorescent staining of Human umbilical vein endothelial cells.<br>anti-collagen III (1:200, PA592066, Invitrogen)- By manufacture western blotting analysis of Mouse brain and pancreas tissue.<br>anti-NF200 (1:50, ab82259, Abcam)- By manufacture immunohistochemistry (Frozen sections) analysis of rat brain and peripheral tissue.<br>anti-S100β (1:100, ab52642, Abcam)- By manufacture immunohistochemistry analysis of rat cerebral cortex tissue.<br>anti–ChAt (1:100, ab181023, Abcam) - By manufacture immunohistochemistry (Formalin/PFA-fixed paraffin-embedded sections) analysis of Human cerebrum tissue sections

Animals and other organisms

Policy information about studies involving animals; ARRIVE guidelines recommended for reporting animal research

Laboratory animals	Housing condition: temperature:21°C; humidity: 35%; dark/light cycle: 12hour/12hour.<br>Animals including : C57 Bl/6J mice, male, 6-8 week old, weight in 25g (Charles River Beijing China)<br>Sprague Dawley rat, male, 8 week old, weight in 300g (Charles River Beijing China)<br>Bama miniature pig, male, weight in 25kg (Laboratory Animal Care Facility of Shanghai Jiao Tong University School of Medicine)
Wild animals	No wild animals were used in study in this section.
Field-collected samples	No field collected samples were used in the study.
Ethics oversight	All the experiments with animals involved in this study were followed by the “Guide for the Care and Use of Laboratory Animals” (NIH, Publication No. 85-23) and “The ARRIVE Guidelines”. All animal experiments were approved by the Animal Experimental Ethics Committee of Ruijin Hospital, Shanghai Jiao Tong University School of Medicine and the protocols related to the experimental animals in this study were compliant with specific ethical regulations.

Note that full information on the approval of the study protocol must also be provided in the manuscript.
